# Metagenomic Analysis Reveals Bacterial and Fungal Diversity and Their Bioremediation Potential From Sediments of River Ganga and Yamuna in India

**DOI:** 10.3389/fmicb.2020.556136

**Published:** 2020-10-16

**Authors:** Bijay Kumar Behera, Hirak Jyoti Chakraborty, Biswanath Patra, Ajaya Kumar Rout, Budheswar Dehury, Basanta Kumar Das, Dhruba Jyoti Sarkar, Pranaya Kumar Parida, Rohan Kumar Raman, Atmakuri Ramakrishna Rao, Anil Rai, Trilochan Mohapatra

**Affiliations:** ^1^Aquatic Environmental Biotechnology & Nanotechnology (AEBN) Division, ICAR-Central Inland Fisheries Research Institute, Kolkata, India; ^2^Department of Chemistry, Technical University of Denmark, Lyngby, Denmark; ^3^Indian Council of Agricultural Research, New Delhi, India; ^4^Centre for Agricultural Bioinformatics, ICAR-Indian Agricultural Statistics Research Institute, New Delhi, India

**Keywords:** metagenomics, Ganga, Yamuna, river sediment, bioremediation

## Abstract

In this study, we report the presence of a microbial community of bioremediation potential in terms of relative abundance and taxonomic biodiversity in sediment samples of river Ganga and Yamuna, India at nine different sites. Metagenomic libraries were constructed using TruSeq Nano DNA Library Prep Kit and sequenced on NextSeq 500 by Illumina Next Generation Sequencing (NGS) technology. Bioremediation bacteria belong to 45 genera with 92 species and fungi belong to 13 genera with 24 species have been classified using Kaiju taxonomical classification. The study revealed that *Proteobacteria* was the most dominant bacterial flora, followed by *Actinobacteria*, *Firmicutes*, and *Deinococcus–Thermus*. PCA analysis revealed that bioremediation bacteria *viz. Streptomyces bikiniensis, Rhodococcus qingshengii, Bacillus aerophilus, Pseudomonas veronii*, etc., were more dominant in highly polluted river stretch as compared to less polluted river stretch. Similarly, the relative abundance of bioremediation fungi *viz. Phanerochaete chrysosporium* and *Rhizopus oryzae*, etc., were significantly correlated with the polluted Kanpur stretch of river Ganga. Several protein domains, which play a pivotal role in bioremediation in the polluted environments, including urea ABC transporter, UrtA, UrtD, UrtE, zinc/cadmium/mercury/lead-transporting ATPase, etc., were identified using protein domain analysis. The protein domains involved in pesticide biodegradation *viz*. P450, short-chain dehydrogenases/reductases (SDR), etc., were also discovered in river sediment metagenomics data. This is the first report on the richness of bioremediation microbial communities in the Ganga and Yamuna riverine ecosystems, highlighting their importance in aquatic pollution management.

## Introduction

Aquatic environmental pollution is a global threat to natural biodiversity and human health (Nicolopoulou-Stamati et al., 2016). With the ever-increasing human population, the use of pesticides and other agro-input has been increased to achieve global food security (Ehrlich and Harte, 2015). Apart from regular use in the agricultural sector, pesticides are used in household purposes to control vector-borne diseases (VBD) like malaria, especially in developing countries (Van den Berg et al., 2012), including India. But the indiscriminate use of pesticides in agriculture and household ultimately results in a significant amount of their residues in an environment which eventually washes out to natural streams like rivers, wetlands, and finally to sea, causing a huge disruption in the natural aquatic biodiversity. It has been reported that pesticide residue found in wetlands, rivers, and sea in substantial amounts can cause the innumerable ill effect to aquatic organisms like endocrine disruption, growth reduction, etc. (Carvalho et al., 2009). Uses of organochlorine (OC) pesticides are banned in many countries for a long, but they are still found in the natural streams, sediments, aquatic flora, and fauna due to their high persistency. Several aquatic ecosystems including rivers, wetlands, and sea were being continuously monitored regularly for assessing the level of OC and their risk assessment on aquatic life (DiScenza et al., 2017; Fair et al., 2018; Unyimadu et al., 2018). Due to their high lipophilicity properties, they are being easily adsorbed on the river sediments and by the aquatic fauna. High concentrations of OC have been reported in the fish, *Drapane africana* (2237–6368 μg/kg) as compared to *Mochokus niloticus* (1006–3288 μg/kg) in the Niger River of Nigeria (Unyimadu et al., 2018). Gene networks involved in the reproduction and immune function of largemouth bass, are reported to disrupt in OC contaminated sites of the Lake Apopka (Martyniuk et al., 2016). Other than OC, the frequently used pesticides found in the natural stream belong to the group organophosphate, carbamates, pyrithroids, etc. Chlorpyrifos and other organophosphate pesticides are reported to impair immune function and structural integrity of the fish *Cyprinus carpio* L. through oxidative stress and apoptosis (Jiao et al., 2017). Lambda-cigalothrine, a pyrithroid insecticide has been reported to cause the alleviation of free amino acid content in the muscle, liver, and brain of fishes in the Alazani River (Dzhikiia et al., 2011).

Because of the toxic effect of pesticidal residues on the native flora and fauna, several physical and chemical methods are being used to eliminate these toxic xenobiotics from natural environments, like landfills, recycling, pyrolysis, etc., However, bioremediation using different microorganisms was found to be the most feasible technique, proved to be worked in many contaminated sites. It was reported that the presence of organophosphate hydrolase (OPH) enzyme in these microorganisms has the capacity of detoxifying them through cleavage of phosphate ester bonds (P-O, P-F, P-CN, and P-S) (Schofield and DiNovo, 2010). The yeast (*Saccharomyces cerevisiae*) capable of hydrolyzing the poorly hydrolyzed P-S class of organophosphate by integrating gene, encoding the wild-type OPH (enhanced variant enzyme S308L-OPH) into the ribosomal operon of the same (Makkar et al., 2013). Similarly, bacterial strain, *Bacillus pumilis*, was reported to be capable of bioremediation of methyl parathion, a P-S type OP pesticide, due to the presence of *opdA* gene (Ali et al., 2012). Several microorganisms belonging to the genera *Bacillus, Brevibacillus, Ochrobactrum, Pseudomonas, Serratia*, and *Sphingobium* are reported to be capable of degrading various pyrithroid pesticides through metabolic activity (Cycon and Piotrowska-Seget, 2016). Several genes with pyrithroid degrading ability were reported *viz. estP* in *Klebsiella* sp. ZD112 (Wu et al., 2006), *pye3* from the metagenome of soil (Li et al., 2008), *pytH* in *Sphingobium* sp. JZ-1 (Wang et al., 2009), *pytZ* and *pytY* (from a genomic library of *Ochrobactrum anthropi* YZ-1) (Zhai et al., 2012). Not only bacteria, but several fungi were also reported with the ability of pesticide remediation through the catabolic or co-metabolic process. Fungi belonging to the genera *Aspergillus, Candida, Cladosporium, Tricoderma*, etc., were reported to degrade different pyrethroid pesticides like cyfluthrin, bifenthrin, deltamethrin, etc. (Cycon and Piotrowska-Seget, 2016).

The natural aquatic ecosystem holds and perishes a good amount of microbial population, which can degrade these toxic xenobiotic residues *in situ*. Therefore, to identify these potential microorganisms and their functional role, metagenomic studies have been conducted extensively in recent years. Metagenomic studies revealed a sediment microbiome in the Deepwater Horizon oil spill and the impact of oil deposition on microbial communities in surface sediments (Mason et al., 2014). Metagenome-assembled genomes (MAGs) revealed the microbial communities of two thermal pools in Kamchatka, Russia (Wilkins et al., 2019). Similarly, the key bacterial species were characterized in the *Daphnia magna* microbiota using shotgun metagenomics (Cooper and Cressler, 2020). Antibiotic Resistance Genes (AMRs) from the sediments of River Yamuna have been identified from the metagenomic study (Das et al., 2020). Using the metagenomics approach, potential microbial community involved in the biodegradation of phenanthrene, diesel, and hexadecane in the mangrove sediment were identified and the degrading bacteria belonged to genera *Bacillus* sp., *Pseudomonas* sp., *Acinetobacter* sp., and *Staphylococcus* sp. (Tiralerdpanich et al., 2018). The metagenomic approach was also utilized to demonstrate the role of IS1071, an insertion element flanks with xenobiotic degradation, in the formation and distribution of bacterial catabolic pathway gene cluster (Dunon et al., 2018). The biodegradation pathway of DDT, HCH, and atrazine in freshwater and marine sediments was analyzed through a metagenomics approach. The study identified 69 genera capable of degrading these persistent pesticides with major populations belonging to genus *Plesiocystis* sp., *Anaerolinea* sp., *Jannaschia* sp., and *Mycobacterium* sp., and found the presence of different genes *viz.* atzB, hdg, and hdt which encode for ethylaminohydrolase, dehalogenase, and hydratase, respectively (Fang et al., 2014). Carbamate pesticide degrading enzyme was also reported by functional metagenomic analysis of rumen samples of Holstein dairy cows (Ufarte et al., 2017). Similarly, the metagenomics approach was utilized to understand the underlying mechanism of biodegradation *in situ* and to predict the degradation potential of microbes in the soil of Queensland, Australia (Jeffries et al., 2018). However, there were not many studies on the occurrence of the native microbial population in the Ganga and Yamuna river ecosystem having bioremediation potential. With this background, the present study accentuates the identification and relative abundance of potential bioremediation microbes capable of degrading pollutants in these river sediments through the metagenomics approach. Furthermore, the study also aims to investigate the diversity and relative abundance of these bioremediation microbes in the different polluted and non-polluted sites of river Ganga and Yamuna.

## Materials and Methods

### Sample Collection and DNA Extraction

**FIGURE 1:**
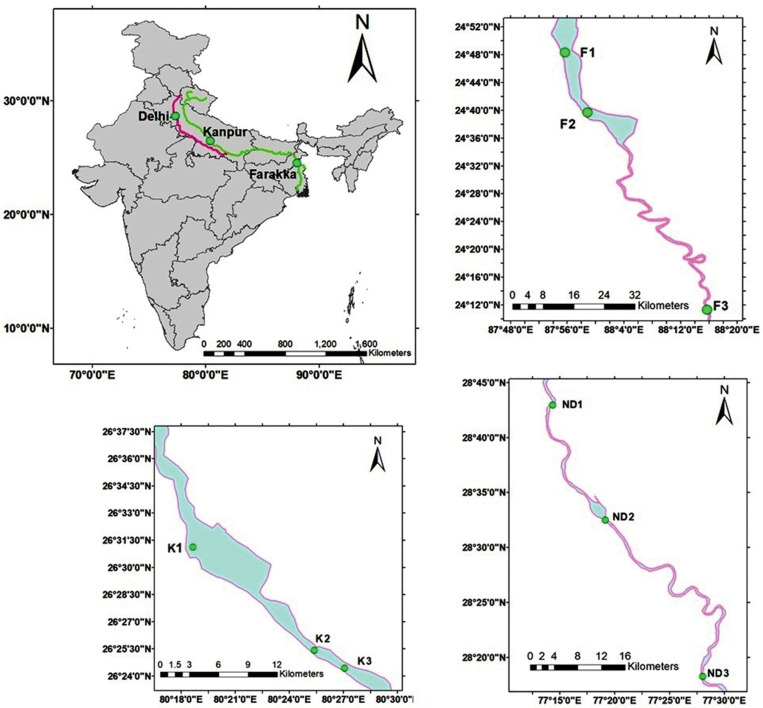
Map showing the sediment sampling sites. Sediments were collected from the river Ganga at six locations namely, K1 (Nawabganj, Kanpur), K2 (Jajmau, Kanpur), K3 (Jana village, Kanpur), F1 (Below Farakka bridge, West Bengal), F2 (Paharghati, West Bengal), and F3 (Lalbag, West Bengal) whereas, three locations from the river Yamuna *viz.* ND1 (Wazaribad, New Delhi), ND2 (Okhla Barrage, New Delhi), and ND3 (Faizupur Khadar, New Delhi).

Sediment and water samples were collected from nine different sites of river Ganga *viz.* Ganga Barrage (K1, N 26°0.858″E 80°19.114″), Jajmau (K2, N 26°25.301″E 80°25.282″), Jana Village (K3, N 26°24.495″E 80°26.904″) near Kanpur, Uttar Pradesh, Farakka Barrage (F1, N 24°47.804″E 87°55.417″), Dhulian (F2, N 24°47.804″E 87°55.417″), Lalbagh (F3, N 29°11.087″E 88°16.079″) near Farakka, West Bengal and three different sites of river Yamuna at stretches *viz.* Wazaribad (ND1, N 28°42.39″E 77°13.57″), Okhla Barrage (ND2, N 28°2.51″E 77°18.30″), Faizupur Khaddar (ND3, N 28°18.43″E 77°27.52″) New Delhi, India during morning hours (8 to 11 AM) (Figure 1). All sediment samples were collected in plastic bags (sterile), sealed, and transported on ice (4°C) and stored at −80°C for metagenomics experiments. Metagenomic DNA from river sediment samples was extracted using a soil gDNA isolation Kit (Nucleospin Soil, Takara). After genomic DNA isolation, the quality was measured in Nanodrop 2000 and Qubit 3.0 Fluorometer and run in Agarose gel. Good quality sediment genomic DNA was used for next-generation library preparation.

### Preparation of 2 × 150 NextSeq 500 Shotgun Libraries

Illumina Trueseq Nano DNA Library Prep Kit was used to prepare paired-end sequencing library. 200 ng of sediment gDNA was fragmented by CovarisM220 to produce a mean fragment allocation of 350 bp. CovarisM220 shearing method generates dsDNA fragments with 3^/^or 5^/^overhangs. All the fragments were then moved to end-repair. In the next step, products were PCR amplified with the index primer as provided in the Kit (TrueSeq DNA Nano Kit-Illumina). The libraries were analyzed in 4200 Tape Station System (Agilent Technologies) after PCR amplification using D1000 Screen tape.

### Cluster Generation, Sequencing, Quality Control, and *de novo* Assembly

The Qubit fluorometric concentration for the libraries was measured and the mean peak size from Agilent Tape Station profile, the PE Illumina libraries were loaded into NextSeq 500 for cluster generation and sequencing. The CLC Genomics Workbench v8.5.1 was used to filter the high-quality reads of each sample and then processed into scaffolds.

### Bioinformatics Analysis of Sediment Metagenome

The Trimmomatic v0.35 was used to process the sequenced raw data to obtain high-quality Clean read and to remove adapter sequences, ambiguous read (reads with unknown nucleotides “N” larger than 5%), and low-quality sequences (reads with more than 10% quality threshold {QV} < 20 phred Score). A minimum length of 100 nucleotides after trimming was applied. The high quality (QV > 20), paired-end reads were used for read assembly. Parameters considered for filtration such as, Adapter trimming; SLIDINGWINDOW: sliding window trimming of 20 bp, cutting once the average quality within the window falls below a threshold of 20; LEADING: cut bases off the start of a read, if bellow a threshold quality of 20; TRAILING: cut bases off the end of a read, if bellow a threshold quality of 20; MINLENGTH: Drop the read if it is below 100 bp length.

The filtered metagenomic reads were used for taxonomical assignment by the Kaiju web server^1^ for identification of bioremediation microbial species in the river sediment metagenome using NR protein database NCBI BLAST nr as reference database (Menzel et al., 2016; Cucio et al., 2018; Verce et al., 2019). Kaiju used Burrows-Wheeler transform algorithm for taxonomic classification on the protein-level. “Greedy” run mode was applied with a minimum match score of 70, and an allowance of five mismatches (Menzel et al., 2016). Further, other webserver/software *viz.* MG RAST and CLC Genomics Workbench v8.5.1 were used for validation of the identified bioremidation microbial communities. To study the phylogenetic relationship among the bioremediation bacterial species found in the river sediment metagenomes, multiple sequence analysis was carried out using MEGA 6 software (Tamura et al., 2013). The Neighbor-Joining method was used to infer evolutionary history (Saitou and Nei, 1987). The Maximum Composite Likelihood method (Tamura et al., 2004) was used to compute evolutionary distances. In this study, 92 identified bioremediation bacterial species, derived from the sediments of the rivers, Ganga and Yamuna were used. Heat map figures were generated using multiple experiment viewer (Mev), a web-based tool for visualizing the clustering of multivariate data (Howe et al., 2010). Following the identification of the bioremediation bacterial gene from metagenomic samples, the set of genes have been considered for ORF prediction. The resultant ORFs were then assessed for the identification of putative or conserved domains.

### Analysis of Physico-Chemical Parameters

The DO (ppm), TDS (ppm), specific conductivity (μS/cm), salinity (%), pH, BOD (ppm), and COD (ppm) of collected river water samples from nine sampling sites were analyzed as per the standard methods of APHA (2012). The pH, specific conductivity (μS/cm), total nitrogen (%), available phosphate (mg/100 g), and organic C (%) of collected river sediment samples from nine sampling sites were also analyzed as per the standard methods of APHA (2012).

### Statistical Analysis

The Principal Component Analysis (PCA) biplot and Scatter Plot Matrix along with correlation values between sampling sites and relative abundance of bioremediation microbes were developed in JMP Pro 10 after standardization of the estimated data. The Diversity index (β-diversity) analysis was carried out using Past software (version 4.03).

## Results

### Generation of Sediment Metagenome Data

Sediment samples were collected from the river Ganga at two locations namely, Kanpur (K1, K2, and K3) and Farakka (F1, F2, and F3), each with three sampling sites. While, from the river Yamuna, sediment samples were collected from the New Delhi stretch (ND1, ND2, and ND3) at three sampling sites. The measurements of isolated metagenomic DNA for quality and quantity have been presented in Table 1. The isolated river sediments metagenomic DNA from these nine sites were analyzed using high-throughput NGS technology and the total number of high quality reads (bp) of each sample collected from Kanpur, Farakka, and New Delhi stretches were 28718955(K1), 33703138(K2), 33887572(K3), 24929338(F1), 29128182(F2), 54496302(F3), 64876611(ND1), 64749798(ND2), and 62670420(ND3), respectively. All the high quality reads obtained from the sediments of different sites were considered for *in silico* analysis to discover the microbial diversity with bioremediation potential. Severe pollution by untreated sewage from the hundreds of tanneries, pharmaceutical industries, municipal waste, chemicals, pesticides, etc., were reported from New Delhi and Kanpur stretches. It was found that some critical pollution parameters (*viz.* BOD, COD, total nitrogen, and phosphate) were higher in these two sites as compared to the less polluted sites at Farraka. The details of the physicochemical parameters of water and sediments collected from these sites are presented in Table 2.

**TABLE 1:** Average concentration of metagenomic DNA isolated from different river sediment samples.

**Sl. No.**	**Sample ID**	**Qubit nucleic acid concn. (ng/μ l)**	**Nano drop OD A_260__/__280_**
1	F1	6.60	1.80
2	F2	6.20	1.83
3	F3	46.00	1.87
4	K1	45.80	1.80
5	K2	80.20	1.85
6	K3	185.60	1.80
7	ND1	227.10	1.83
8	ND2	186.50	1.82
9	ND3	203.20	1.84

**TABLE 2:** Physiochemical parameters of water and sediment at different sampling sites of river Ganga and Yamuna.

	**Pollution Parameters**	**River ganga**						**River yamuna**		
		**Kanpur**			**Farraka**			**New Delhi**		
		**Up side of ganga barrage (K1)**	**Jajmau (K2)**	**Jana village (K2)**	**Farakka barrage (F1)**	**Dhulian (F2)**	**Lalbagh (F3)**	**Wazaribad (ND1)**	**Okhla barrage (ND2)**	**Faizupur khaddar(ND3)**
Water	DO (ppm)	5.01	4.38	4.16	6.34	6.39	6.77	1.9	3.1	2.8
	TDS (ppm)	170.4	316	227	95.5	94.3	96.5	342.5	466.3	356.15
	Specific Conductivity (μS/cm)	356	641	469	200.7	198.3	222.6	425.3	895	651.23
	Salinity (%)	0.17	0.31	0.23	0.09	0.09	0.09	0.23	0.14	0.15
	pH	8.10	7.84	7.9	8.20	7.78	7.82	7.98	7.65	7.237
	BOD (ppm)	1.0	6.0	6.2	0.2	0.8	0.5	5.1	7.9	4.23
	COD (ppm)	2.0	9.0	8.0	0.16	1.8	0.8	5.9	11.23	8.56
Sediment	pH	7.2	7.8	8.1	7.01	7.5	7.6	7.5	7.9	7.8
	Specific Conductivity (μS/cm)	0.09	0.54	0.89	0.06	0.12	0.11	0.15	0.65	0.35
	Total Nitrogen (%)	0.06	0.09	0.07	0.04	0.05	0.05	0.04	0.06	0.08
	Available Phosphate (mg/100g)	4.0	5.8	5.2	2.6	5.4	3.2	4.2	8.2	6.8
	Organic C (%)	0.10	0.32	0.19	0.35	0.43	0.21	0.09	0.3	0.47

### Taxonomical Classification of Sediment Metagenomes

Bioremediation bacteria belong to 45 genera with 92 species and fungi belong to 13 genera with 24 species have been classified using Kaiju taxonomical classification, MG RAST and CLC Genomics Workbench v8.5.1. A large number of bioremediation microbes with their relative abundance were identified from the sediment samples of river Ganga and Yamuna based on taxonomical classification (Supplementary Table S1). Twelve *Bacillus* sp., thirteen *Pseudomonas* sp., four *Burkholderia* sp., four *Streptomyces* sp., four *Rhodococcus* sp. three *Sphingobium* sp., *Acinetobacter* sp., *Aeromonas* sp., two *Sphingomonas* sp., *Stenotrophomonas* sp., *Ochrobactrum* sp., *Geobacter* sp., *Enterobacter* sp., *Desulfovibrio* sp. and more other bacterial species have been identified from the metagenomics data set. Among the phylums, it was found that *Proteobacteria* was most abundant, followed by *Actinobacteria*, *Firmicutes*, and *Deinococcus-Thermus*. Similarly, many bioremediation fungi *viz.* six *Aspergillus* sp., three *Phanerochaete* sp., two *Bjerkandera* sp., two *Pleurotus* sp., two *Rhizopus* sp., two *Trametes* sp., *Clonostachys* sp., *Coprinus* sp., *Exophiala* sp., *Fusarium* sp., *Mucor* sp., *Penicillium* sp. and *Trichoderma* sp. were also identified (Supplementary Table S2).

### Phylogenetic Analysis of Bioremediation Bacteria

**FIGURE 2:**
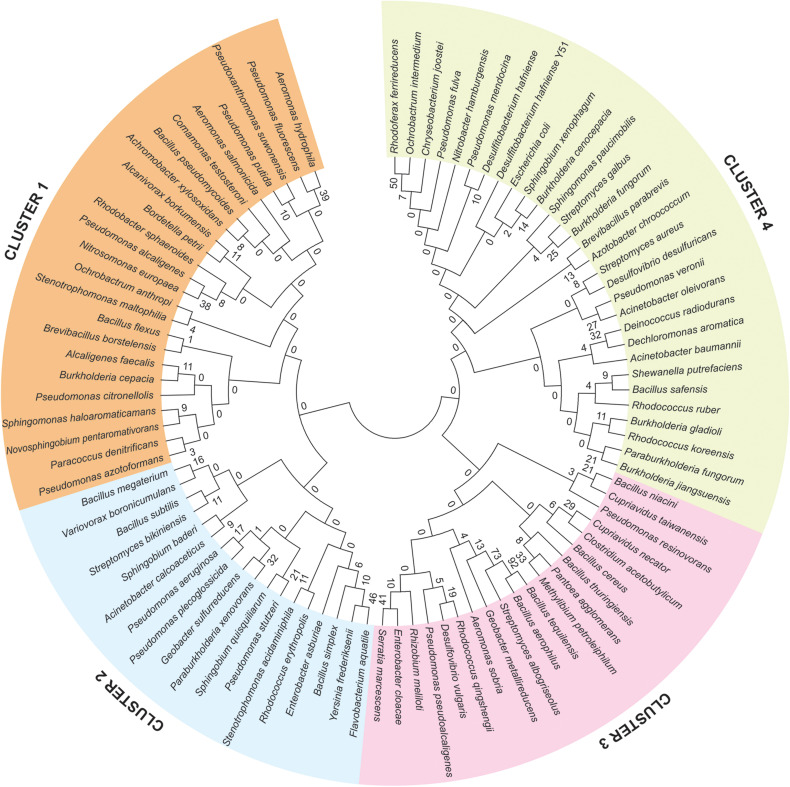
Phylogenetic analysis of 92 identified genome of bioremediation bacterial species, derived from the sediments of the river Ganga and Yamuna. Phylogenetic cladogram clearly demarcated that, all the identified bacterial species shaped four different clusters represented in different colors and the bootstrap values are provided at the nodes of the phylogenetic tree.

To understand the evolutionary relationship among the 92 identified genomes of bioremediation bacterial species, a Multiple Sequence Analysis (MSA) using MEGA 6 has been carried out. Based on the MSA, phylogenetic relationships were established. Out of four clusters, in cluster 1, *Pseudomonas fluorescens* and *Aeromonas hydrophila* lies in one clade with the highest bootstrap value. Similarly, *Flavobacterium aquatile* and *Yersinia frederiksenii* are closely related to each other in Cluster 2, while, *Bacillus tequilensis* and *Bacillus aerophilus* are phylogenetically closely related in Cluster 3. *Rhodoferax ferrireducens* and *Ochrobactrum intermedium* are very close to each other in Cluster 4 (Figure 2).

### Microbial Diversity Analysis

**FIGURE 3:**
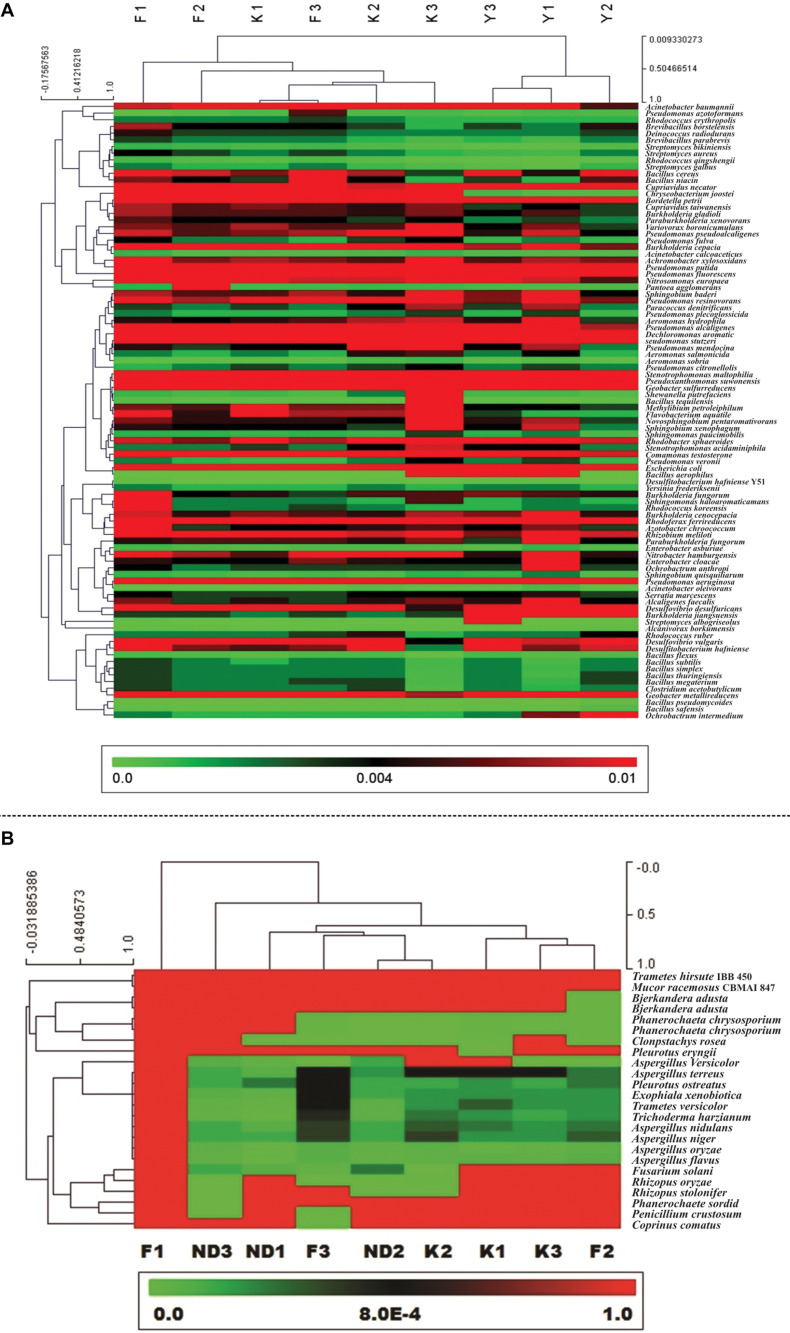
Relative abundance Heat map of identified 92 bacterial species **(A)** and 24 fungal species **(B)** having bioremediation potential from nine different sediment metagenomes of river Ganga and Yamuna.

In the classified metagenomics data, a total of 92 bioremediation bacterial species from the 45 genera were considered for diversity analysis. Heat map analysis showed the clear distinction in the relative abundance of bioremediation bacteria between Kanpur and Farakka sediment samples which might be due to the higher level of pollution at Kanpur stretch of the river Ganga. The river Yamuna at New Delhi stretch was found to be highly polluted and contaminated by industrial and municipal swages. Heat map analysis portrayed a clear demarcation on the species relative abundance among nine different sites in the bacterial metagenomics data (Figure 3A) and 24 species of fungi (Figure 3B). Relative abundance analysis revealed that the various *Streptomyces* sp. were present in higher proportion in sediment samples of river Ganga (Farakka and Kanpur stretches) compare to river Yamuna. *Streptomyces albogriseolus, Streptomyces aureus*, and *Streptomyces bikiniensis* species were present in relatively high proportion at Farakka stretch of the river Ganga (Supplementary Table S1). From these metagenomics data, two *Rhodococcus* species (*Rhodococcus erythropolis* and *Rhodococcus qingshengii*) were found in higher abundance in Farakka stretch as compared to Yamuna stretch. *Cupriavidus necator, Cupriavidus taiwanensis* and *Paraburkholderia xenovorans* were also found higher abundance in Farakka stretch. *Pseudomonas citronellolis* was found in higher abundance in the Kanpur stretch as compared to Farakka and New Delhi stretches. Similarly, *Flavobacterium aquatile, Methylibium petroleiphilum, Pantoea agglomerans, Bordetella petrii, Flavobacterium aquatile, Methylibium petroleiphilum* were found higher in Kanpur stretch. *Chryseobacterium joostei* and *Desulfovibrio desulfuricans* were highly abundant in the New Delhi stretch as compared to Kanpur and Farakka stretches (Supplementary Table S1).

Among the bioremediation fungi, *Aspergillus terreus, Trichoderma harzianum*, and *Trametes versicolor* are found higher quantities in Kanpur stretch as compared to Farakka and New Delhi stretches. *Aspergillus versicolor* and *Fusarium solani* are observed in higher quantities in New Delhi stretch of the river Yamuna compared to the Kanpur and Farraka stretch of the river Ganga (Supplementary Table S2). The diversity index between nine sampling sites (β-diversity) with heat map was analyzed (Whittaker method) and presented in Supplementary Figures S1, S2. The β-diversity of bioremediation bacteria between F1 and ND3 was highest (0.027). Similarly, the β-diversity of bioremediation fungus was found to be the highest (0.379) between K1 and ND3.

### PCA Analysis

**FIGURE 4:**
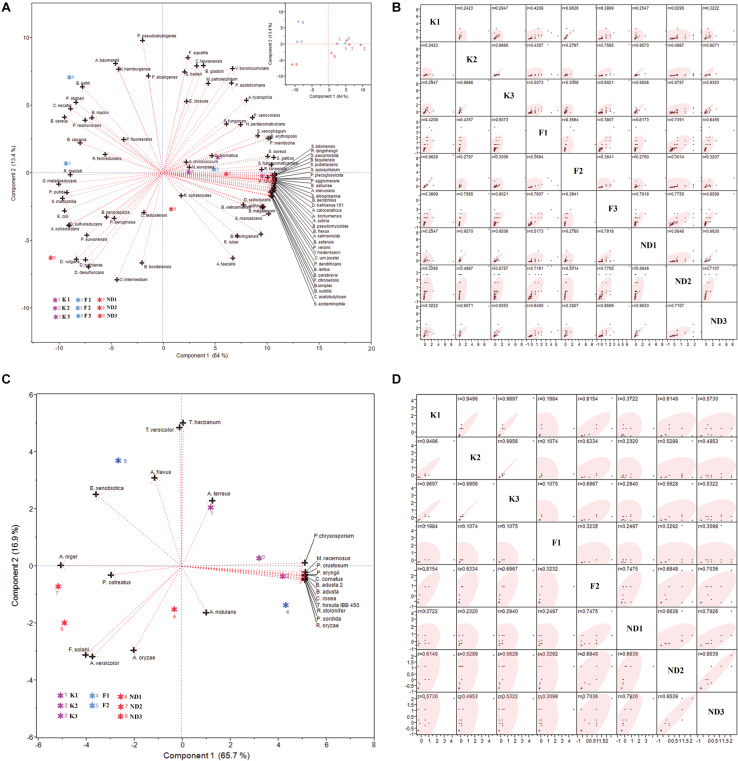
PCA biplot and Scatter Plot Matirx of relative abundance of bioremediation bacteria (**A** and **B**, respectively) and fungi (**C** and **D**, respectively) found at the test sites. PC1 and PC2 together could explain 77.4% and 82.6% of variability in relative abundance of bacteria and fungi, respectively.

The relative abundance of 92 bacterial and 24 fungal species at nine different study sites was analyzed by PCA and correlation between sampling sites was observed in the Scatter Plot Matrix. The PCA biplot of bacterial relative abundance showed that the first two components together could explain 77.4% (PC1, 64% and PC2, 13.4%) variability in the relative abundance data and showed that, majority of the bioremediation bacteria are having high relative abundance at the polluted stretch of river Ganga i.e., Kanpur stretch and river Yamuna i.e., New Delhi stretch (Figure 4A). The Scatter Plot Matrix (Figure 4B) depicted that, some sites were correlated based on the relative abundance of bioremediation bacteria *viz.* K2 and K3 (r = 0.97); ND1 and ND3 (r = 0.96) and F2 and K1(r = 0.96). However, some sites were not found to be correlated with any other sites *viz.* F1, F3, and ND2.

Similarly, the PCA biplot of fungal relative abundance (PC1, 65.7%, and PC2, 16.9%) showed that the majority of the bioremediation fungi are having high relative abundance at the polluted stretch of river Ganga i.e., Kanpur stretch (Figure 4C). It was interesting to note that, the relative abundances of bioremediation fungi were found to zero at the site F1 and the sites F2 and F3 were not found correlated. The Scatter Plot Matrix (Figure 4D) also depicted that, Kanpur sites (K1, K2, and K3) were highly correlated (r > 0.94) so was ND2 and ND3 (r > 0.95).

### Functional Metagenomics-Protein Domain Analysis

ORF’s were predicted from the identified genomes of bioremediation bacterial species, considered for conserved domain (CD) analysis. The CD-search analysis revealed that an immense number of domains related to bioremediation has been found in the sediment metagenome of these rivers. Protein domains related to bioremediation such as ABC transporter (cl25403, cd03214, TIGR03407, cd03297, TIGR02789, TIGR02770, TIGR03411, TIGR03410, cd03260, PRK10419, cd00625, and cl28564) is involved in the transport of a wide variety of different compounds, like molybdenum, nickel, arsenic and more complex organic molecules were found in the sediment metagenomes of river Ganga and Yamuna. Further, other transporter protein domain like, zinc/cadmium/mercury/lead-transporting ATPase (PRK11033), iron-dicitrate transporter (PRK11231), ferric transporter (PRK11432), cobalt transporter (PRK13647), nitrate transporter (TIGR01184), potassium transporter (COG2205), phosphonate/organophosphate ester transporter (PRK09984), ABC-type bacteriocin transporter (TIGR01193), P-type heavy metal-transporting ATPase (cd07546), which play a significant role in bioremediation have also been identified. In addition to that, several hydrolases like Ab-hydrolase, Metallo-dependent hydrolases, N-ethylammeline chlorohydrolase, which play a considerable role in bioremediation, were identified. The Cytochrome P450s domain which is haem-thiolate proteins and several Haloacid Dehalogenase like Hydrolases (HAD) (cd04302, cl21460, cd01427, pfam00702, TIGR01549, cd07512, cd16417, pfam13419) which play an active role in the oxidative degradation of various compounds, degradation of environmental toxins/mutagens and pesticide bioremediation has also been identified. Gene ontology (GO) analysis of Cytochrome P450s reveals that, P450s domain involved in the molecular function and biological process pathways for iron ion binding (GO:0003674), pigment biosynthetic process (GO:0008150), heme-binding (GO:0003674), and oxidoreductase activity (GO:0003674 and GO:0008150). Similarly, GO analysis of all the other identified domains has been carried out and given in Supplementary Figures S3–S6. All the domains related to bioremediation along with their accession number, super-family, and producing organism name has been tabulated in the Supplementary Table S3.

## Discussion

The present metagenomics study identified the potential bioremediation microbes in the sediments of river Ganga and Yamuna and investigates the correlation between microbial relative abundance and diversity with polluted and non-polluted sites of these rivers. The lists of identified bioremediation microbes with target pollutants are given in Supplementary Tables S4, S5. In recent times, bioremediation of several toxic chemicals reported throughout the world. Sediments from rivers and lagoons of the Southern Gulf of Mexico were used for the biodegradation of hexadecane (Garcia-Cruz et al., 2018). The proteins of *Brevibacillus brevis* respond to Triphenyl phosphate and provide novel insights into the biodegradation process of bacteria under adverse environmental conditions (Wei et al., 2018). A previous study revealed that the bacterial isolates of a stream in Manaus-Amazon could bioremediate chromium-polluted ecosystems. These bacteria were classified in 10 genera *viz. Micrococcus* sp., *Proteus* sp., *Acinetobacter* sp., *Acidovorax* sp., *Bacillus* sp., *Enterobacter* sp., *Comamonas* sp., *Alicycliphilus* sp., *Serratia* sp., and *Vagococcus* sp. (Teles et al., 2018).

The present research was designed to identify bioremediation bacteria and fungi species of commercial importance and their relations with different riverine ecosystems using high-throughput sequencing and computational metagenomics. The sensitive taxonomical classification was used for the identification of microbial community biodiversity and their functions (Mendes et al., 2017). A large number of bioremediation bacteria and fungal species (Bacteria: 45 genera and 92 species; fungi: 13 genera and 24 species) (Supplementary Tables S1, S2) were found including pesticide degrading domains in the Ganga and Yamuna river sediments.

To investigate the evolutionary correlations among the identified bioremediation bacterial genome, a phylogenetic tree was constructed which showed the majority of the species are similar throughout the evolution. The phylogenetic tree of all the identified bioremediation species was configured in four different clusters. In Cluster 1, *Pseudomonas fluorescens* and *Aeromonas hydrophila* lies in one clade with the highest bootstrap value. The *Bacillus tequilensis* and *Bacillus aerophilus* are phylogenetically closely related in Cluster 3. The phylogenetic analysis based on the 16S rRNA gene of 181 type strains of *Bacillus* species and related taxa constituted nine clusters. The study revealed that *Bacillus* was not a monophyletic group. *B. subtilis* was in Group 1. Group 4 corresponds to thermophiles, Group 6 to halophilic or halotolerant bacilli, and Group 8 to alkaliphilic bacilli (Wang and Sun, 2009).

Relative abundance study showed that bioremediation bacteria of different genera were equally distributed among the three locations; however, few species dominate in one location over others, *viz. Flavobacterium aquatile, Methylibium petroleiphilum, Pantoea agglomerans, Pseudomonas citronellolis* were highly abundant in Kanpur location of river Ganga as compared to other locations. These location-specific changes of microbial diversity in the river sediments might be due to differential physicochemical properties and pollution level of the collected sediments. The present results also suggested that the relative abundance of bioremediation microbial communities was significantly correlated with polluted sites at river Ganga and Yamuna. The study revealed that polluted sites at New Delhi stretch of Yamuna and Kanpur stretch of Ganga were having a higher relative abundance of bioremediation bacteria and fungi as compared to less polluted Farakka sites.

Farakka stretch of the river Ganga was found to be less polluted (Samanta, 2013) whereas, the Kanpur stretch of Ganga and the New Delhi stretch of the river Yamuna were reported to be severely polluted due to the release of untreated metropolitan swages, factory effluents, etc. (Sehgal et al., 2012; Malik and Tauler, 2013). High concentrations of malathion (2.618 μg/l) and γ-HCH (0.259 μg/l) were detected in the surface water collected from the Ganga riverine ecosystem at Kanpur stretch. From the potable water samples in Delhi, Organochlorine pesticides, mainly isomers of hexachlorohexane, dichloro-diphenyl-trichloroethane, endrin, endosulfan, aldrin, dieldrin, and heptachlore were identified. It had been found α and β isomers of endosulfan residues in the Yamuna river (Agarwal et al., 2015). In the present investigation, the differential physicochemical parameters might be responsible for differences in the relative abundance of bioremediation microbes at different locations in the river Ganga and Yamuna. In the present study, a higher relative abundance of *Bordetella petrii* in Kanpur and Farakka was found as compared to the Yamuna. For that reason, it was postulated that pollution makes differences in the relative abundance of bioremediation bacteria species among different locations in the river Ganga and Yamuna. The β-diversity analysis also revealed that diversity indices of bioremediation bacteria and fungi of Farakka samples are different from the polluted sites of New Delhi.

Pesticide degrading bacteria identified in the present study are important as naturally occurring microbes can be manipulated to degrade highly toxic and carcinogenic compounds. Several microbes like Acephate degrading bacteria, *Enterobacter asburiae, Bacillus cereus, Pantoea agglomerans* was identified from sediments of the river Ganga and Yamuna, and these bacteria are reported earlier for pesticide decomposition by Ramya et al., 2016. Fipronil degrading *Acinetobacter calcoaceticus* and *Acinetobacter oleivorans* (Uniyal et al., 2016) were also found from the present sediment metagenomics at all locations. *Pseudomonas putida* and *Rhodococcus* sp. which degrade both the endosulfan isomers through oxidative and hydrolytic pathways were also found (Singh et al., 2017). Endosulfan degrading bacterial strains *Pusillimonas* sp. JW2 and *Bordetella petrii* NS were reported from polluted water and soil environments (Kong et al., 2018).

Bioremediation fungi *Burkholderia jiangsuensis* was reported to possess methyl parathion hydrolase (MPH) gene (bjmpd) (Liu et al., 2014). The present experiments found a significantly higher relative abundance of *Burkholderia jiangsuensis* in the Farakka stretch, whereas the same was not found at New Delhi stretch of river Yamuna. *Cupriavidus necator* was found to be involved in the degradation of aromatic compounds through 2, 3-dioxygenase pathway (Berezina et al., 2015). *Cupriavidus necator* was also found significantly higher quantities in Farakka locations in the present investigation. Highly competent chlorpyrifos degrading strain of *Cupriavidus taiwanensis* (Wang et al., 2015) was also found in higher proportion at Farakka locations.

Bioremediation fungi, *Aspergillus terreus*, found in the present study was reported to expresses Feruloyl esterases (FAEs) which is the key enzyme for biodegradation of lignocelluloses and hydrolysis of ester linkages between hemicellulose and lignin (Zhang et al., 2015). The study revealed that chlorfenvinphos could be degraded by *Aspergillus fumigatus*, *Penicillium citrinum*, *Aspergillus terreus*, and *Trichoderma harzianum* (Oliveira et al., 2015), and lots of these species were reported from the metagenomics samples. *Phanerochaete chrysosporium*, *Aspergillus awamori*, *Aspergillus flavus*, *Trichoderma viride* found to be involved in heavy metal bioremediation like Pb, Cd, Cr, and Ni (Joshi et al., 2011).

*Aspergillus nidulans* isolated from arsenic-contaminated soil was found to have the arsenic adsorption potential to be 84.35% (Maheswari and Murugesan, 2009). *Aspergillus versicolor* was reported to degrade antibiotic triclosan in simulated wastewater and semi-synthetic media (Tastan and Donmez, 2015). *Exophiala xenobiotica* was found to be dominant black yeast present in the car gasoline tanks, able to use toluene as a carbon source (Isola et al., 2013). *Fusarium solani* was reported to tolerate caffeine concentration of 8 g/L (Nayak et al., 2013). It is reported that *Trametes versicolor* (3000 UL^–1^) could produce laccase and *Pleurotus ostreatus* (2700 UL^–1^) showed the ability to degrade PAHs, phenanthrene (PHE) and pyrene (PYR) (Rosales et al., 2013). In our study, *Aspergillus terreus, Trichoderma harzianum*, and *Trametes versicolor* found higher quantities in Kanpur locations compared to Farakka and New Delhi stretches. *Aspergillus versicolor* and *Fusarium solani* were discovered in higher quantities in the New Delhi stretch of river Yamuna compared to the river Ganga.

PCA has been widely used to interpret significant bioremediation species from the experimental data set (Zitko, 1994; Vega et al., 1998; Shrestha and Kazama, 2007). The present analysis showed that the relative abundance of bacterial and fungal species of bioremediation potential is better correlated with the polluted sites of the river as compared to less polluted sites i.e., Farakka stretch. The study also revealed the location-wise clustering of data for Kanpur and New Delhi stretches of river Ganga and Yamuna due to a high correlation between pollution sites and relative abundance of microbes. Bioremediation fungal species showed a more prominent effect through a strong correlation with polluted sites *viz.* K2 and K3.

In the present metagenome, urea ABC transporter ATP binding protein Urtd gene in *Alcaligenes faecalis*, *Bacillus cereus, Rhodococcus qingshengii*, UrtE gene in *Alcaligenes faecalis* and *Bacillus cereus* bacteria were found in Farakka sample. In Kanpur stretch, UrtA gene was found in *Ochrobactrum anthropi*, UrtD, UrtE gene in *Serratia marcescens*. All members of this protein family ABC transporter ATP-binding subunits or gene clusters are expressed, producing the urea permease and connected with urea transport and metabolism. The role of these genes or domain in the bioremediation of urea can be supported by the previous finding (Valladares et al., 2002) where it was suggested that the involvement of a transporter of this superfamily in urea scavenging by some cyanobacteria in natural environments.

Another domain cd08419 which is a C-terminal substrate-binding of LysR-type transcriptional regulator (CbbR) of RubisCO operon, involved in carbon dioxide fixation was identified (Supplementary Table S3). The versatile function of various metal ion transport, amino acid transport, nickel, nitrate, phosphate, molybdenum, cobalt, Mn2^+^/Zn2^+^ transport, zinc/cadmium/mercury/lead-transporting ATPase, etc., found in the present metagenome were likely to play an active role in bioremediation process. In bacteria *Pseudomonas putida*, P450 was reported to be involved in Hexachlorobenzene and Pentachlorobenzene metabolism earlier (Scott et al., 2008). The Oxidoreductase gene was also reported in *Pseudomonas* sp. LBr, *Agrobacterium* strain T10 participates in Glyphosphate biodegradation (Scott et al., 2008). In the present study, enzymes from two different microbes, *Burkholderia xenovorans* and *Flavobacterium aquatile* were discovered which are actively involved in pesticide bioremediation.

The Metagenomic sequences have been submitted to the NCBI-SRA database under the Accession Nos: Kanpur Samples K1 (SRP190174), K2 (SRP190175), K3 (SRP189880), Farakka Samples, F1 (SRP191076), F2 (SRP191079), F3 (SRP191075), and New Delhi Samples, ND1 (SRP191073), ND2 (SRP191080), and ND3 (SRP191499) respectively.

## Conclusion

This is the first report on the identification of bioremediation microbes in sediments of the river Ganga and Yamuna in India, using a metagenomic approach. The present study unraveled the in-depth insights on the relative abundance of important native bacteria and fungi convoluted in bioremediation in these rivers and their functional properties for possible biotechnological applications. The identified *Nitrobacter humburgensis* could reduce heavy metals like zinc, cadmium, mercury, and lead. Similarly, *Rhodobacter sphaeroides* could reduce arsenic, *Shewanella putrefaciens* for iron, *Bacillus cereus* for molybdenum and *Alcaligenes faecalis* for PAHs. The identified fungus like *Aspergillus flaves* could reduce lead, cadmium, chromium, and nickel, and *Aspergillus nidulans* for arsenic, etc., It was found that these bioremediation bacteria and fungi were more abundant in the sediments of a polluted stretch of the river. Several identified protein domains, which are actively involved in environmental bioremediation processes, could be explored for genome-scale engineering for industrial applications in the future.

## Data Availability Statement

The datasets presented in this study can be found in online repositories. The names of the repository/repositories and accession number(s) can be found in the article/ Supplementary Material.

## Author Contributions

BKB designed the metagenomics study and also collected samples for this research. HJC, BP and AKR performed bioinformatics data analysis. DJS and RR performed statistical analysis. BKB, BP, HJC, BD, AKR, DJS, PKP, ARR, AR, BKD, and TM wrote the manuscript. All authors participated in revision of the final manuscript for submission.

## Conflict of Interest

The authors declare that the research was conducted in the absence of any commercial or financial relationships that could be construed as a potential conflict of interest.

## Footnotes

1 http://kaiju.binf.ku.dk
