# Supplementary material for: Metagenomic Analysis Reveals Bacterial and Fungal Diversity and Their Bioremediation Potential From Sediments of River Ganga and Yamuna in India
Source: Front Microbiol. 2020 Oct 16;11:556136. doi: 10.3389/fmicb.2020.556136 (PMC7596357; doi:10.3389/fmicb.2020.556136)
Supplement: Supplementary file 1 [file Data_Sheet_1.docx]

|  | **K1** | **K2** | **K3** | **F1** | **F2** | **F3** | **ND1** | **ND2** | **ND3** |
| --- | --- | --- | --- | --- | --- | --- | --- | --- | --- |
| **K1** | 0 | 0 | 0.005525 | 0.011111 | 0.005525 | 0 | 0.010989 | 0.005465 | 0.016575 |
| **K2** | 0 | 0 | 0.005525 | 0.011111 | 0.005525 | 0 | 0.010989 | 0.005465 | 0.016575 |
| **K3** | 0.005525 | 0.005525 | 0 | 0.01676 | 0.011111 | 0.0055249 | 0.005525 | 0.010989 | 0.011111 |
| **F1** | 0.011111 | 0.011111 | 0.01676 | 0 | 0.01676 | 0.011111 | 0.022222 | 0.016575 | 0.027933 |
| **F2** | 0.005525 | 0.005525 | 0.011111 | 0.01676 | 0 | 0.0055249 | 0.016575 | 0.010989 | 0.022222 |
| **F3** | 0 | 0 | 0.005525 | 0.011111 | 0.005525 | 0 | 0.010989 | 0.005465 | 0.016575 |
| **ND1** | 0.010989 | 0.010989 | 0.005525 | 0.022222 | 0.016575 | 0.010989 | 0 | 0.005465 | 0.0055249 |
| **ND2** | 0.005465 | 0.005465 | 0.010989 | 0.016575 | 0.010989 | 0.0054645 | 0.005465 | 0 | 0.010989 |
| **ND3** | 0.016575 | 0.016575 | 0.011111 | 0.027933 | 0.022222 | 0.016575 | 0.005525 | 0.010989 | 0 |

**Supplementary Figure S1:** The β diversity analysis of bioremediation bacteria in the different sampling sites using Whittkar method. The red colour intensity signifies the higher dissimilarity in the availability of bioremediation bacteria in different sampling sites. Similarly, the green colour intensity signifies higher similarity in the availability of bioremediation bacteria in different sampling sites. K1 (Nawabganj, Kanpur), K2 (Jajmau, Kanpur), K3 (Jana village, Kanpur), F1 (Below Farakka bridge, West Bengal), F2 (Paharghati, West Bengal) and F3 (Lalbag, West Bengal) whereas, three locations from the river Yamuna *viz.* ND1 (Wazaribad, New Delhi), ND2 (Okhla Barrage, New Delhi) and ND3 (Faizupur Khadar, New Delhi).

|  | **K1** | **K2** | **K3** | **F1** | **F2** | **F3** | **ND1** | **ND2** | **ND3** |
| --- | --- | --- | --- | --- | --- | --- | --- | --- | --- |
| **K1** | 0 | 0.14286 | 0.12 | 1 | 0.14286 | 0.22581 | 0.2 | 0.17241 | 0.37931 |
| **K2** | 0.14286 | 0 | 0.18519 | 1 | 0.2 | 0.15152 | 0.18519 | 0.032258 | 0.22581 |
| **K3** | 0.12 | 0.18519 | 0 | 1 | 0.11111 | 0.2 | 0.16667 | 0.14286 | 0.28571 |
| **F1** | 1 | 1 | 1 | 0 | 1 | 1 | 1 | 1 | 1 |
| **F2** | 0.14286 | 0.2 | 0.11111 | 1 | 0 | 0.21212 | 0.18519 | 0.16129 | 0.35484 |
| **F3** | 0.22581 | 0.15152 | 0.2 | 1 | 0.21212 | 0 | 0.2 | 0.11765 | 0.23529 |
| **ND1** | 0.2 | 0.18519 | 0.16667 | 1 | 0.18519 | 0.2 | 0 | 0.14286 | 0.21429 |
| **ND2** | 0.17241 | 0.032258 | 0.14286 | 1 | 0.16129 | 0.11765 | 0.14286 | 0 | 0.1875 |
| **ND3** | 0.37931 | 0.22581 | 0.28571 | 1 | 0.35484 | 0.23529 | 0.21429 | 0.1875 | 0 |

**Supplementary Figure S2:**  The β diversity analysis of bioremediation fungus in the different sampling sites using Whittkar method. The red colour intensity signifies the higher dissimilarity in the availability of bioremediation fungus in different sampling sites. Similarly, the green colour intensity signifies higher similarity in the availability of bioremediation fungus in different sampling sites.


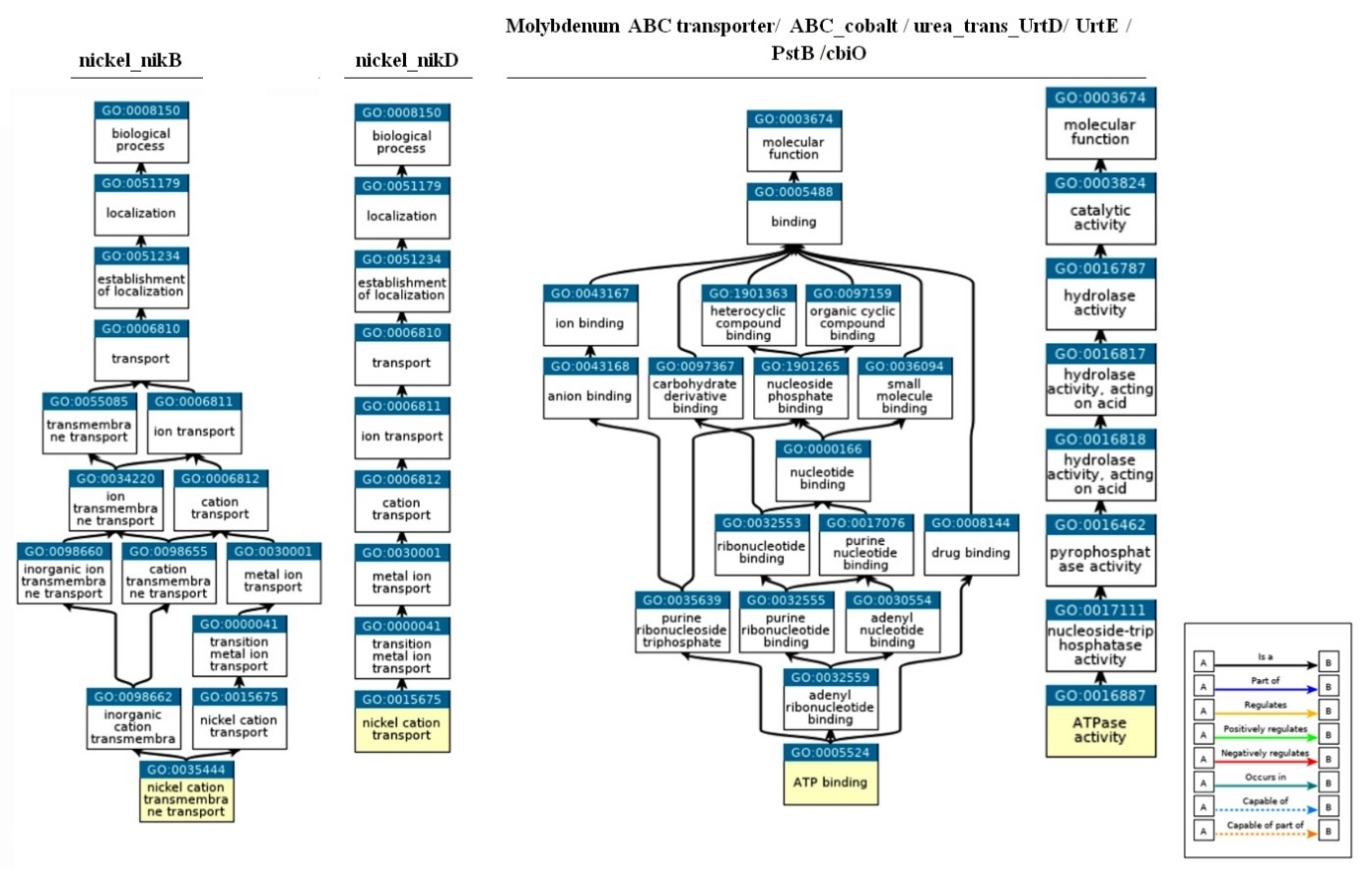


**Supplementary Figure S3:** Gene Ontology (GO) analysis of identified bioremediation Domains showing Ancestor Chart of nickel cation transmembrane transport, nickel cation transport, ATP binding and ATPase activity.

**Supplementary Figure
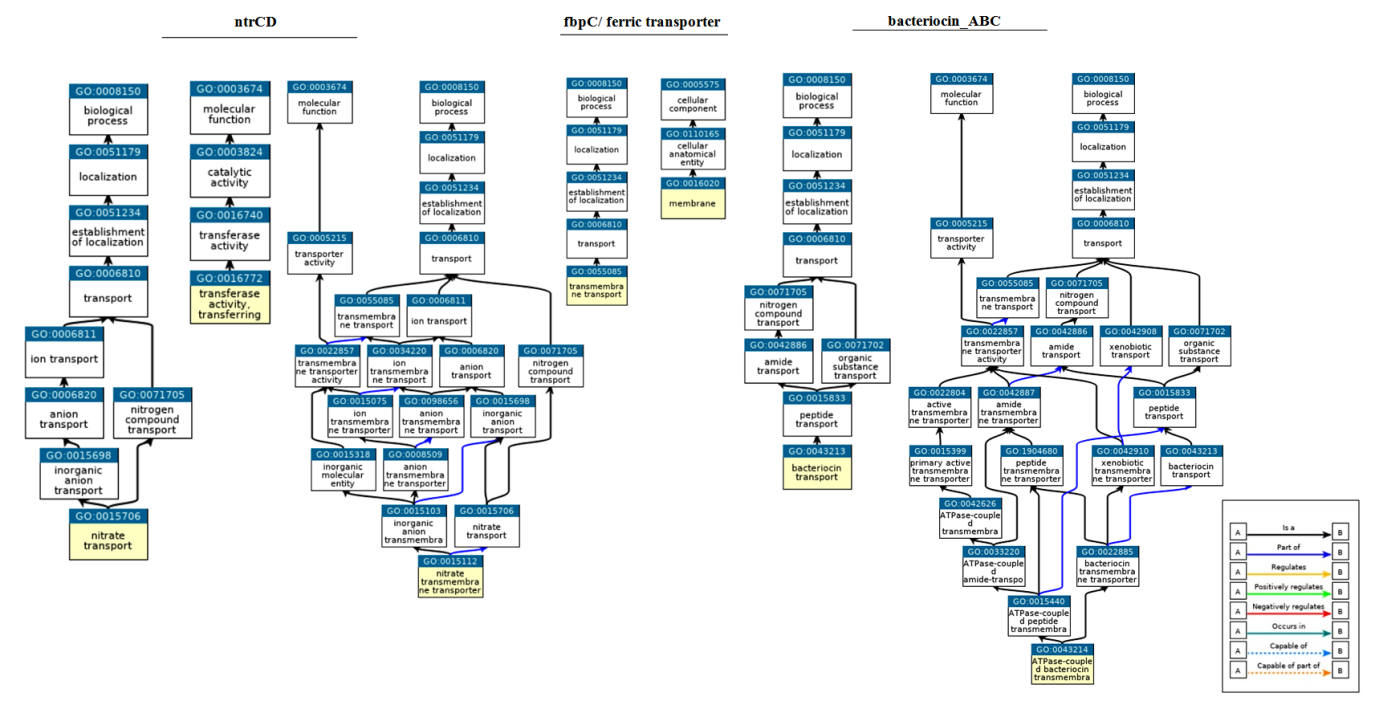
S4:** Gene Ontology (GO) analysis of identified bioremediation Domains showing Ancestor Chart of nitrate transport, transferase activity, nitrate transmembrane transport, bacteriocin transport and ATPase -couple bacteriocin transport activity.


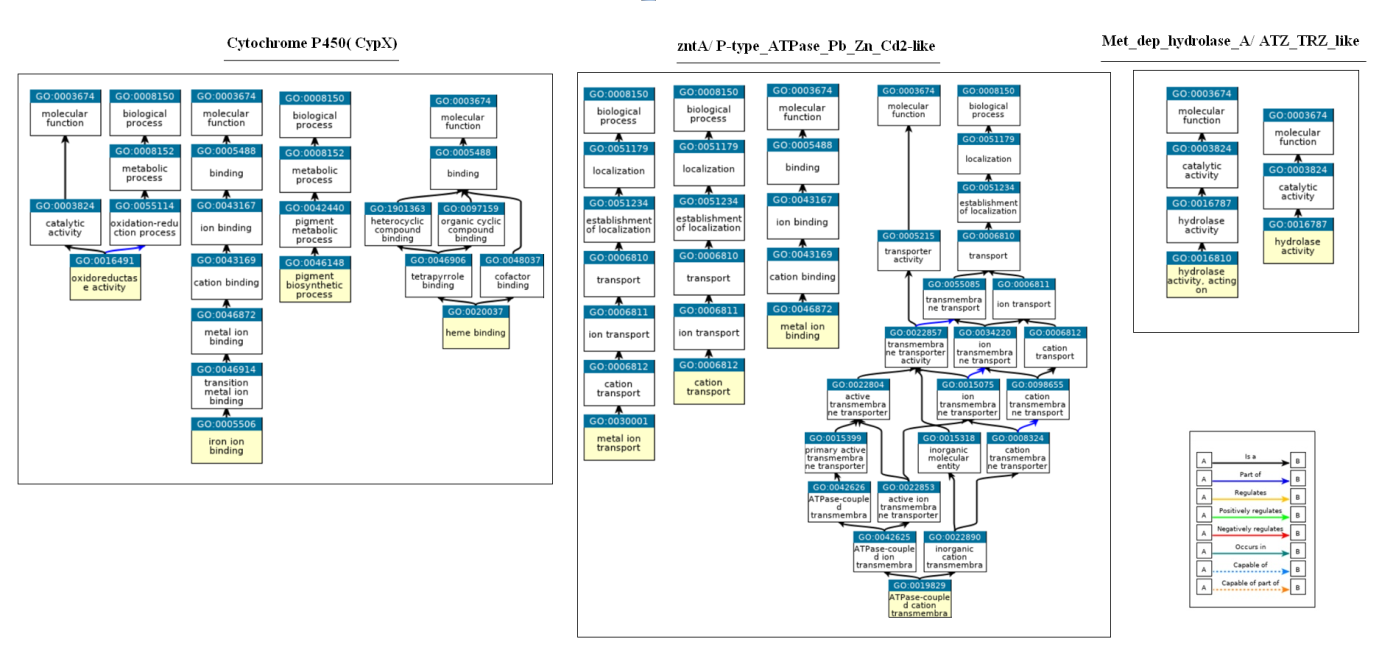


**Supplementary Figure S5:** Gene Ontology (GO) analysis of identified bioremediation Domains showing Ancestor Chart of oxidoreductase activity , iron ion binding activity, pigment biosynthetic process, heme binding, metal ion binding, ATPase -couple cation transmembrane transport and hydrolase activity etc .

**Supplementary Figure**
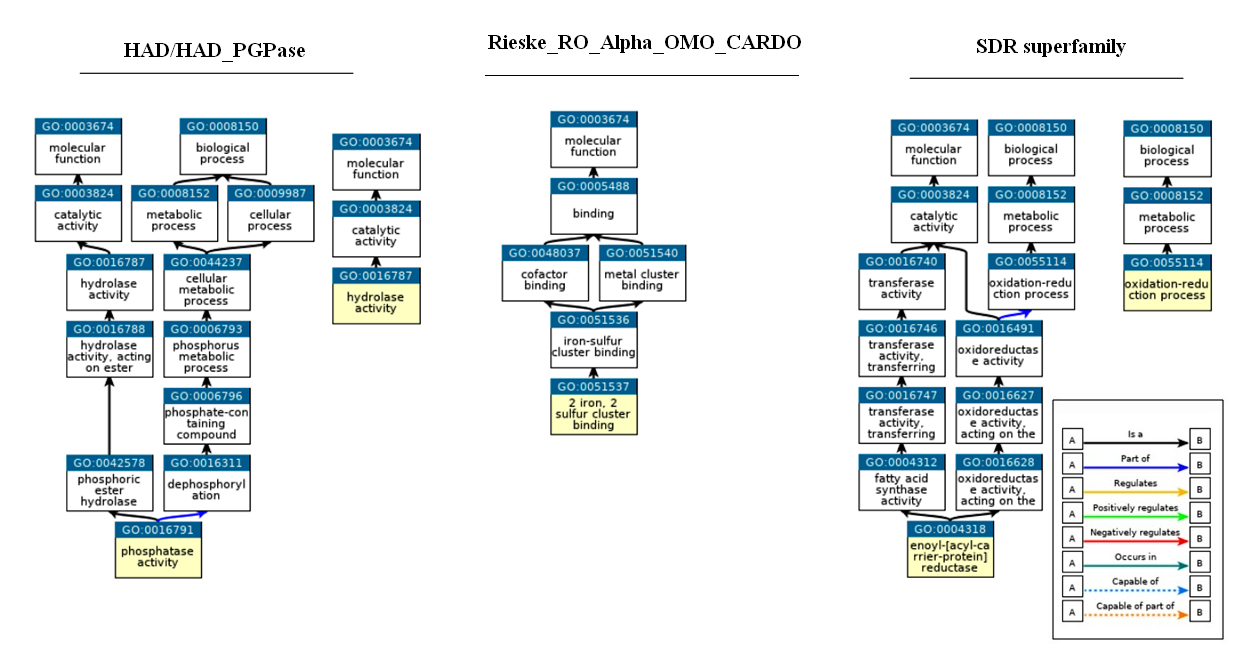
**S6:** Gene Ontology (GO) analysis of identified bioremediation Domains showing Ancestor Chart of phosphatase activity, hydrolase activity, 2 iron 2 sulfur cluster binding, enoyl-acyl-carrier protein reductase and oxidation -reduction process etc.

**Supplementary Table S1**. Relative abundance of bioremediation bacteria found in different locations of the river Ganga and Yamuna

| **Sl. No** | **Genus** | **Name of species** | **K1** | **K2** | **K3** | **Average** | **SD** | **F1** | **F2** | **F3** | **Average** | **SD** | **ND1** | **ND2** | **ND3** | **Average** | **SD** |
| --- | --- | --- | --- | --- | --- | --- | --- | --- | --- | --- | --- | --- | --- | --- | --- | --- | --- |
| 1 | Achromobacter | *A. xylosoxidans* | 0.008 | 0.006 | 0.01 | 8.0E-03 | 2.0E-03 | 0.01 | 0.007 | 0.007 | 8.0E-03 | 1.7E-03 | 0.008 | 0.007 | 0.006 | 7.0E-03 | 1.0E-03 |
| 2 | Acinetobacter | *A. baumannii* | 0.01 | 0.02 | 0.03 | 2.0E-02 | 1.0E-02 | 0.009 | 0.01 | 0.03 | 1.6E-02 | 1.2E-02 | 0.01 | 0.006 | 0.01 | 8.7E-03 | 2.3E-03 |
|  |  | *A. calcoaceticus* | 0.0005 | 0.0006 | 0.0009 | 6.7E-04 | 2.1E-04 | 0.0009 | 0.0004 | 0.0005 | 6.0E-04 | 2.6E-04 | 0.0005 | 0.0004 | 0.0005 | 4.7E-04 | 5.8E-05 |
|  |  | *A. oleivorans* | 0.00009 | 0.0003 | 0.0002 | 2.0E-04 | 1.1E-04 | 0.0001 | 0.00005 | 0.0001 | 8.3E-05 | 2.9E-05 | 0.0006 | 0.00007 | 0.0002 | 2.9E-04 | 2.8E-04 |
| 3 | Aeromonas | *A. hydrophila* | 0.005 | 0.008 | 0.009 | 7.3E-03 | 2.1E-03 | 0.005 | 0.004 | 0.006 | 5.0E-03 | 1.0E-03 | 0.01 | 0.003 | 0.005 | 6.0E-03 | 3.6E-03 |
|  |  | *A. salmonicida* | 0.002 | 0.005 | 0.006 | 4.3E-03 | 2.1E-03 | 0.002 | 0.001 | 0.001 | 1.3E-03 | 5.8E-04 | 0.004 | 0.001 | 0.002 | 2.3E-03 | 1.5E-03 |
|  |  | *A. sobria* | 0.0002 | 0.0006 | 0.0008 | 5.3E-04 | 3.1E-04 | 0.0002 | 0.0002 | 0.0002 | 2.0E-04 | 0.0E+00 | 0.0004 | 0.0002 | 0.0002 | 2.7E-04 | 1.2E-04 |
| 4 | Alcaligenes | *A. faecalis* | 0.005 | 0.005 | 0.007 | 5.7E-03 | 1.2E-03 | 0.007 | 0.003 | 0.003 | 4.3E-03 | 2.3E-03 | 0.01 | 0.004 | 0.005 | 6.3E-03 | 3.2E-03 |
| 5 | Alcanivorax | *A. borkumensis* | 0.00004 | 0.0001 | 0.0001 | 8.0E-05 | 3.5E-05 | 0.0001 | 0.0001 | 0.00007 | 9.0E-05 | 1.7E-05 | 0.00008 | 0.0001 | 0.0001 | 9.3E-05 | 1.2E-05 |
| 6 | Azotobacter | *A. chroococcum* | 0.004 | 0.005 | 0.006 | 5.0E-03 | 1.0E-03 | 0.01 | 0.003 | 0.004 | 5.7E-03 | 3.8E-03 | 0.007 | 0.003 | 0.004 | 4.7E-03 | 2.1E-03 |
| 7 | Bacillus | *B. aerophilus* | 0.000009 | 0.000006 | 0 | 5.0E-06 | 4.6E-06 | 0.00003 | 0.000009 | 0.00001 | 1.6E-05 | 1.2E-05 | 0 | 0.00003 | 0 | 1.0E-05 | 1.7E-05 |
|  |  | *B. cereus* | 0.007 | 0.008 | 0.003 | 6.0E-03 | 2.6E-03 | 0.02 | 0.009 | 0.02 | 1.6E-02 | 6.4E-03 | 0.005 | 0.01 | 0.01 | 8.3E-03 | 2.9E-03 |
|  |  | *B. flexus* | 0.0003 | 0.0005 | 0.00006 | 2.9E-04 | 2.2E-04 | 0.0005 | 0.0004 | 0.0005 | 4.7E-04 | 5.8E-05 | 0.0003 | 0.0005 | 0.0006 | 4.7E-04 | 1.5E-04 |
|  |  | *B. megaterium* | 0.002 | 0.003 | 0.0006 | 1.9E-03 | 1.2E-03 | 0.003 | 0.002 | 0.003 | 2.7E-03 | 5.8E-04 | 0.0009 | 0.003 | 0.002 | 2.0E-03 | 1.1E-03 |
|  |  | *B. niacini* | 0.003 | 0.004 | 0.001 | 2.7E-03 | 1.5E-03 | 0.007 | 0.004 | 0.01 | 7.0E-03 | 3.0E-03 | 0.001 | 0.006 | 0.003 | 3.3E-03 | 2.5E-03 |
|  |  | *B. pseudomycoides* | 0.00007 | 0.0001 | 0.00003 | 6.7E-05 | 3.5E-05 | 0.0001 | 0.00007 | 0.00008 | 8.3E-05 | 1.5E-05 | 0.00003 | 0.0002 | 0.00008 | 1.0E-04 | 8.7E-05 |
|  |  | *B. safensis* | 0.0001 | 0.0001 | 0.00006 | 8.7E-05 | 2.3E-05 | 0.0002 | 0.0001 | 0.0001 | 1.3E-04 | 5.8E-05 | 0.0001 | 0.0004 | 0.0002 | 2.3E-04 | 1.5E-04 |
|  |  | *B.simplex* | 0.002 | 0.002 | 0.0007 | 1.6E-03 | 7.5E-04 | 0.003 | 0.002 | 0.002 | 2.3E-03 | 5.8E-04 | 0.0008 | 0.002 | 0.002 | 1.6E-03 | 6.9E-04 |
|  |  | *B. subtilis* | 0.001 | 0.002 | 0.0007 | 1.2E-03 | 6.8E-04 | 0.003 | 0.002 | 0.002 | 2.3E-03 | 5.8E-04 | 0.0006 | 0.002 | 0.002 | 1.5E-03 | 8.1E-04 |
|  |  | *B. tequilensis* | 0.0002 | 0.0001 | 0.01 | 3.4E-03 | 5.7E-03 | 0.0003 | 0.0002 | 0.0002 | 2.3E-04 | 5.8E-05 | 0.00004 | 0.0002 | 0.00009 | 1.1E-04 | 8.2E-05 |
|  |  | *B. thuringiensis* | 0.002 | 0.002 | 0.0006 | 1.5E-03 | 8.1E-04 | 0.003 | 0.002 | 0.002 | 2.3E-03 | 5.8E-04 | 0.0007 | 0.003 | 0.002 | 1.9E-03 | 1.2E-03 |
|  |  | *B. lentus* | 0.002 | 0.002 | 0.0005 | 1.5E-03 | 8.7E-04 | 0.003 | 0.002 | 0.002 | 2.3E-03 | 5.8E-04 | 0.0007 | 0.002 | 0.001 | 1.2E-03 | 6.8E-04 |
| 8 | Bordetella | *B. petrii* | 0.04 | 0.02 | 0.04 | 3.3E-02 | 1.2E-02 | 0.04 | 0.03 | 0.03 | 3.3E-02 | 5.8E-03 | 0.02 | 0.01 | 0.01 | 1.3E-02 | 5.8E-03 |
| 9 | Brevibacillus | *B. borstelensis* | 0.004 | 0.003 | 0.001 | 2.7E-03 | 1.5E-03 | 0.008 | 0.004 | 0.004 | 5.3E-03 | 2.3E-03 | 0.002 | 0.005 | 0.003 | 3.3E-03 | 1.5E-03 |
|  |  | *B. parabrevis* | 0.002 | 0.001 | 0.0006 | 1.2E-03 | 7.2E-04 | 0.003 | 0.002 | 0.002 | 2.3E-03 | 5.8E-04 | 0.0006 | 0.002 | 0.001 | 1.2E-03 | 7.2E-04 |
| 10 | Burkholderia | *B. cenocepacia* | 0.007 | 0.005 | 0.008 | 6.7E-03 | 1.5E-03 | 0.009 | 0.005 | 0.006 | 6.7E-03 | 2.1E-03 | 0.01 | 0.005 | 0.007 | 7.3E-03 | 2.5E-03 |
|  |  | *B. cepacia* | 0.01 | 0.01 | 0.02 | 1.3E-02 | 5.8E-03 | 0.02 | 0.01 | 0.01 | 1.3E-02 | 5.8E-03 | 0.01 | 0.007 | 0.008 | 8.3E-03 | 1.5E-03 |
|  |  | *B. vietnamiensis* | 0.005 | 0.007 | 0.008 | 6.7E-03 | 1.5E-03 | 0.007 | 0.005 | 0.005 | 5.7E-03 | 1.2E-03 | 0.01 | 0.004 | 0.007 | 7.0E-03 | 3.0E-03 |
|  |  | *B. gladioli* | 0.006 | 0.005 | 0.007 | 6.0E-03 | 1.0E-03 | 0.008 | 0.006 | 0.006 | 6.7E-03 | 1.2E-03 | 0.006 | 0.003 | 0.004 | 4.3E-03 | 1.5E-03 |
| 11 | Chryseobacterium | *C. um joostei* | 0 | 0 | 0 | 0.0E+00 | 0.0E+00 | 0 | 0 | 0 | 0.0E+00 | 0.0E+00 | 0.0004 | 0.0004 | 0.0007 | 5.0E-04 | 1.7E-04 |
| 12 | Clostridium | *C. acetobutylicum* | 0.002 | 0.003 | 0.0005 | 1.8E-03 | 1.3E-03 | 0.003 | 0.002 | 0.002 | 2.3E-03 | 5.8E-04 | 0.001 | 0.002 | 0.002 | 1.7E-03 | 5.8E-04 |
| 13 | Comamonas | *C. testosteroni* | 0.02 | 0.03 | 0.06 | 3.7E-02 | 2.1E-02 | 0.02 | 0.01 | 0.01 | 1.3E-02 | 5.8E-03 | 0.04 | 0.009 | 0.04 | 3.0E-02 | 1.8E-02 |
| 14 | Cupriavidus | *C. necator* | 0.01 | 0.009 | 0.01 | 9.7E-03 | 5.8E-04 | 0.02 | 0.02 | 0.02 | 2.0E-02 | 0.0E+00 | 0.02 | 0.01 | 0.01 | 1.3E-02 | 5.8E-03 |
|  |  | *C. taiwanensis* | 0.007 | 0.005 | 0.007 | 6.3E-03 | 1.2E-03 | 0.008 | 0.006 | 0.006 | 6.7E-03 | 1.2E-03 | 0.004 | 0.003 | 0.003 | 3.3E-03 | 5.8E-04 |
| 15 | Dechloromonas | *D. aromatica* | 0.08 | 1 | 0.6 | 5.6E-01 | 4.6E-01 | 0.06 | 0.09 | 0.08 | 7.7E-02 | 1.5E-02 | 0.6 | 0.04 | 0.2 | 2.8E-01 | 2.9E-01 |
| 16 | Deinococcus | *D. radiodurans* | 0.003 | 0.002 | 0.002 | 2.3E-03 | 5.8E-04 | 0.005 | 0.003 | 0.003 | 3.7E-03 | 1.2E-03 | 0.002 | 0.003 | 0.002 | 2.3E-03 | 5.8E-04 |
| 17 | Desulfitobacterium | *D. hafniense* | 0.006 | 0.01 | 0.002 | 6.0E-03 | 4.0E-03 | 0.01 | 0.007 | 0.007 | 8.0E-03 | 1.7E-03 | 0.007 | 0.009 | 0.01 | 8.7E-03 | 1.5E-03 |
|  |  | *D. hafniense Y51* | 0.000009 | 0.00009 | 0.00001 | 3.6E-05 | 4.6E-05 | 0.00009 | 0.00002 | 0.00002 | 4.3E-05 | 4.0E-05 | 0.00007 | 0.00002 | 0.0001 | 6.3E-05 | 4.0E-05 |
| 18 | Desulfovibrio | *D. desulfuricans* | 0.01 | 0.01 | 0.005 | 8.3E-03 | 2.9E-03 | 0.02 | 0.01 | 0.01 | 1.3E-02 | 5.8E-03 | 0.05 | 0.02 | 0.03 | 3.3E-02 | 1.5E-02 |
|  |  | *D. vulgaris* | 0.01 | 0.02 | 0.004 | 1.1E-02 | 8.1E-03 | 0.02 | 0.01 | 0.01 | 1.3E-02 | 5.8E-03 | 0.009 | 0.02 | 0.02 | 1.6E-02 | 6.4E-03 |
| 19 | Enterobacter | *E. asburiae* | 0.0004 | 0.0005 | 0.0005 | 4.7E-04 | 5.8E-05 | 0.0007 | 0.0005 | 0.0005 | 5.7E-04 | 1.2E-04 | 0.0009 | 0.0004 | 0.0003 | 5.3E-04 | 3.2E-04 |
|  |  | *E. cloacae* | 0.004 | 0.005 | 0.004 | 4.3E-03 | 5.8E-04 | 0.005 | 0.004 | 0.007 | 5.3E-03 | 1.5E-03 | 0.01 | 0.004 | 0.004 | 6.0E-03 | 3.5E-03 |
| 20 | Escherichia | *E. coli* | 0.02 | 0.02 | 0.03 | 2.3E-02 | 5.8E-03 | 0.02 | 0.02 | 0.02 | 2.0E-02 | 0.0E+00 | 0.03 | 0.02 | 0.03 | 2.7E-02 | 5.8E-03 |
| 21 | Flavobacterium | *F. aquatile* | 0.01 | 0.008 | 0.03 | 1.6E-02 | 1.2E-02 | 0.01 | 0.005 | 0.008 | 7.7E-03 | 2.5E-03 | 0.001 | 0.001 | 0.003 | 1.7E-03 | 1.2E-03 |
| 22 | Geobacter | *G. metallireducens* | 0.06 | 0.06 | 0.008 | 4.3E-02 | 3.0E-02 | 0.07 | 0.06 | 0.04 | 5.7E-02 | 1.5E-02 | 0.02 | 0.04 | 0.03 | 3.0E-02 | 1.0E-02 |
|  |  | *G. sulfurreducens* | 0.03 | 0.04 | 0.1 | 5.7E-02 | 3.8E-02 | 0.04 | 0.03 | 0.02 | 3.0E-02 | 1.0E-02 | 0.02 | 0.03 | 0.03 | 2.7E-02 | 5.8E-03 |
| 23 | Methylibium | *M. petroleiphilum* | 0.01 | 0.007 | 0.03 | 1.6E-02 | 1.3E-02 | 0.007 | 0.006 | 0.007 | 6.7E-03 | 5.8E-04 | 0.004 | 0.003 | 0.004 | 3.7E-03 | 5.8E-04 |
| 24 | Nitrobacter | *N. hamburgensis* | 0.006 | 0.01 | 0.01 | 8.7E-03 | 2.3E-03 | 0.01 | 0.008 | 0.01 | 9.3E-03 | 1.2E-03 | 0.02 | 0.005 | 0.005 | 1.0E-02 | 8.7E-03 |
| 26 | Nitrosomonas | *N. europaea* | 0.3 | 0.009 | 0.02 | 1.1E-01 | 1.6E-01 | 0.02 | 0.3 | 0.009 | 1.1E-01 | 1.6E-01 | 0.009 | 0.006 | 0.01 | 8.3E-03 | 2.1E-03 |
| 27 | Novosphingobium | *N. pentaromativorans* | 0.005 | 0.004 | 0.02 | 9.7E-03 | 9.0E-03 | 0.007 | 0.005 | 0.005 | 5.7E-03 | 1.2E-03 | 0.008 | 0.003 | 0.005 | 5.3E-03 | 2.5E-03 |
| 28 | Ochrobactrum | *O. anthropi* | 0.003 | 0.003 | 0.003 | 3.0E-03 | 5.3E-19 | 0.004 | 0.002 | 0.003 | 3.0E-03 | 1.0E-03 | 0.01 | 0.003 | 0.003 | 5.3E-03 | 4.0E-03 |
|  |  | *O. intermedium* | 0.001 | 0.001 | 0.001 | 1.0E-03 | 0.0E+00 | 0.002 | 0.001 | 0.001 | 1.3E-03 | 5.8E-04 | 0.007 | 0.03 | 0.002 | 1.3E-02 | 1.5E-02 |
| 29 | Pantoea | *P. agglomerans* | 0.0007 | 0.0008 | 0.0008 | 7.7E-04 | 5.8E-05 | 0.001 | 0 | 0.0007 | 5.7E-04 | 5.1E-04 | 0.0005 | 0.0005 | 0.0006 | 5.3E-04 | 5.8E-05 |
| 30 | Paraburkholderia | *P. fungorum* | 0.006 | 0.005 | 0.007 | 6.0E-03 | 1.0E-03 | 0.005 | 0.006 | 0.006 | 5.7E-03 | 5.8E-04 | 0.01 | 0.004 | 0.003 | 5.7E-03 | 3.8E-03 |
|  |  | *P. xenovorans* | 0.004 | 0.003 | 0.004 | 3.7E-03 | 5.8E-04 | 0.006 | 0.004 | 0.004 | 4.7E-03 | 1.2E-03 | 0.003 | 0.002 | 0.002 | 2.3E-03 | 5.8E-04 |
| 31 | Paracoccus | *P. denitrificans* | 0.003 | 0.004 | 0.008 | 5.0E-03 | 2.6E-03 | 0.003 | 0.002 | 0.002 | 2.3E-03 | 5.8E-04 | 0.008 | 0.002 | 0.003 | 4.3E-03 | 3.2E-03 |
| 32 | Pseudomonas | *P. aeruginosa* | 0.03 | 0.07 | 0.08 | 6.0E-02 | 2.6E-02 | 0.03 | 0.02 | 0.03 | 2.7E-02 | 5.8E-03 | 0.2 | 0.03 | 0.08 | 1.0E-01 | 8.7E-02 |
|  |  | *P. alcaligenes* | 0.02 | 0.05 | 0.05 | 4.0E-02 | 1.7E-02 | 0.01 | 0.01 | 0.02 | 1.3E-02 | 5.8E-03 | 0.06 | 0.008 | 0.01 | 2.6E-02 | 2.9E-02 |
|  |  | *P. azotoformans* | 0.0005 | 0.0005 | 0.0006 | 5.3E-04 | 5.8E-05 | 0.0001 | 0.0003 | 0.006 | 2.1E-03 | 3.4E-03 | 0.0006 | 0.0005 | 0.0004 | 5.0E-04 | 1.0E-04 |
|  |  | *P. citronellolis* | 0.003 | 0.003 | 0.004 | 3.3E-03 | 5.8E-04 | 0.001 | 0.002 | 0.002 | 1.7E-03 | 5.8E-04 | 0.003 | 0.002 | 0.002 | 2.3E-03 | 5.8E-04 |
|  |  | *P. fluorescens* | 0.1 | 0.02 | 0.04 | 5.3E-02 | 4.2E-02 | 0.03 | 0.02 | 0.02 | 2.3E-02 | 5.8E-03 | 0.02 | 0.01 | 0.02 | 1.7E-02 | 5.8E-03 |
|  |  | *P. fulva* | 0.002 | 0.003 | 0.004 | 3.0E-03 | 1.0E-03 | 0.004 | 0.002 | 0.001 | 2.3E-03 | 1.5E-03 | 0.002 | 0.0009 | 0.0009 | 1.3E-03 | 6.4E-04 |
|  |  | *P. mendocina* | 0.004 | 0.01 | 0.01 | 8.0E-03 | 3.5E-03 | 0.005 | 0.003 | 0.004 | 4.0E-03 | 1.0E-03 | 0.008 | 0.002 | 0.004 | 4.7E-03 | 3.1E-03 |
|  |  | *P. plecoglossicida* | 0.001 | 0.002 | 0.003 | 2.0E-03 | 1.0E-03 | 0.002 | 0.001 | 0.001 | 1.3E-03 | 5.8E-04 | 0.003 | 0.0008 | 0.001 | 1.6E-03 | 1.2E-03 |
|  |  | *P. pseudoalcaligenes* | 0.007 | 0.01 | 0.01 | 9.0E-03 | 1.7E-03 | 0.009 | 0.006 | 0.008 | 7.7E-03 | 1.5E-03 | 0.009 | 0.004 | 0.005 | 6.0E-03 | 2.6E-03 |
|  |  | *P. putida* | 0.02 | 0.01 | 0.02 | 1.7E-02 | 5.8E-03 | 0.02 | 0.01 | 0.01 | 1.3E-02 | 5.8E-03 | 0.02 | 0.01 | 0.01 | 1.3E-02 | 5.8E-03 |
|  |  | *P. resinovorans* | 0.01 | 0.01 | 0.02 | 1.3E-02 | 5.8E-03 | 0.01 | 0.008 | 0.01 | 9.3E-03 | 1.2E-03 | 0.02 | 0.007 | 0.007 | 1.1E-02 | 7.5E-03 |
|  |  | *P. stutzeri* | 0.02 | 0.04 | 0.04 | 3.3E-02 | 1.2E-02 | 0.02 | 0.01 | 0.02 | 1.7E-02 | 5.8E-03 | 0.03 | 0.01 | 0.02 | 2.0E-02 | 1.0E-02 |
|  |  | *P. veronii* | 0.0008 | 0.002 | 0.004 | 2.3E-03 | 1.6E-03 | 0.002 | 0.001 | 0.0008 | 1.3E-03 | 6.4E-04 | 0.003 | 0.0009 | 0.002 | 2.0E-03 | 1.1E-03 |
| 33 | Pseudoxanthomonas | *P. suwonensis* | 0.04 | 0.03 | 0.1 | 5.7E-02 | 3.8E-02 | 0.02 | 0.02 | 0.02 | 2.0E-02 | 0.0E+00 | 0.02 | 0.03 | 0.03 | 2.7E-02 | 5.8E-03 |
| 34 | Sinorhizobium | *R. meliloti* | 0.01 | 0.009 | 0.01 | 9.7E-03 | 5.8E-04 | 0.02 | 0.01 | 0.01 | 1.3E-02 | 5.8E-03 | 0.02 | 0.009 | 0.007 | 1.2E-02 | 7.0E-03 |
| 35 | Rhodobacter | *R. sphaeroides* | 0.02 | 0.02 | 0.07 | 3.7E-02 | 2.9E-02 | 0.009 | 0.007 | 0.008 | 8.0E-03 | 1.0E-03 | 0.04 | 0.009 | 0.01 | 2.0E-02 | 1.8E-02 |
| 36 | Rhodococcus | *R. erythropolis* | 0.002 | 0.002 | 0.001 | 1.7E-03 | 5.8E-04 | 0.002 | 0.002 | 0.003 | 2.3E-03 | 5.8E-04 | 0.001 | 0.001 | 0.001 | 1.0E-03 | 0.0E+00 |
|  |  | *R. koreensis* | 0.002 | 0.002 | 0.001 | 1.7E-03 | 5.8E-04 | 0 | 0.002 | 0.003 | 1.7E-03 | 1.5E-03 | 0.002 | 0.002 | 0.001 | 1.7E-03 | 5.8E-04 |
|  |  | *R. qingshengii* | 0.0003 | 0.0003 | 0.0002 | 2.7E-04 | 5.8E-05 | 0.0007 | 0.0004 | 0.0005 | 5.3E-04 | 1.5E-04 | 0.0001 | 0.0002 | 0.0002 | 1.7E-04 | 5.8E-05 |
|  |  | *R. ruber* | 0.002 | 0.005 | 0.001 | 2.7E-03 | 2.1E-03 | 0.002 | 0.002 | 0.003 | 2.3E-03 | 5.8E-04 | 0.002 | 0.004 | 0.002 | 2.7E-03 | 1.2E-03 |
| 37 | Rhodoferax | *R. ferrireducens* | 0.03 | 0.03 | 0.05 | 3.7E-02 | 1.2E-02 | 0.09 | 0.02 | 0.02 | 4.3E-02 | 4.0E-02 | 0.09 | 0.01 | 0.04 | 4.7E-02 | 4.0E-02 |
| 38 | Serratia | *S. marcescens* | 0.003 | 0.004 | 0.004 | 3.7E-03 | 5.8E-04 | 0.004 | 0.003 | 0.003 | 3.3E-03 | 5.8E-04 | 0.005 | 0.003 | 0.004 | 4.0E-03 | 1.0E-03 |
| 39 | Shewanella | *S. putrefaciens* | 0.0008 | 0.002 | 0.01 | 4.3E-03 | 5.0E-03 | 0.0009 | 0.0006 | 0.0005 | 6.7E-04 | 2.1E-04 | 0.0007 | 0.0004 | 0.0007 | 6.0E-04 | 1.7E-04 |
| 40 | Sphingobium | *S. baderi* | 0.006 | 0.005 | 0.02 | 1.0E-02 | 8.4E-03 | 0.008 | 0.006 | 0.008 | 7.3E-03 | 1.2E-03 | 0.02 | 0.004 | 0.007 | 1.0E-02 | 8.5E-03 |
|  |  | *S. quisquiliarum* | 0.0005 | 0.0005 | 0.001 | 6.7E-04 | 2.9E-04 | 0.0009 | 0.0007 | 0.0007 | 7.7E-04 | 1.2E-04 | 0.002 | 0.0004 | 0.0009 | 1.1E-03 | 8.2E-04 |
|  |  | *S. xenophagum* | 0.004 | 0.003 | 0.02 | 9.0E-03 | 9.5E-03 | 0.005 | 0.004 | 0.004 | 4.3E-03 | 5.8E-04 | 0.009 | 0.002 | 0.004 | 5.0E-03 | 3.6E-03 |
| 41 | Sphingomonas | *S. haloaromaticamans* | 0.002 | 0.001 | 0.006 | 3.0E-03 | 2.6E-03 | 0 | 0.002 | 0.002 | 1.3E-03 | 1.2E-03 | 0.002 | 0.001 | 0.001 | 1.3E-03 | 5.8E-04 |
|  |  | *S. paucimobilis* | 0.0009 | 0.0009 | 0.003 | 1.6E-03 | 1.2E-03 | 0.001 | 0.001 | 0.001 | 1.0E-03 | 0.0E+00 | 0.002 | 0.0006 | 0.001 | 1.2E-03 | 7.2E-04 |
| 42 | Stenotrophomonas | *S. acidaminiphila* | 0.003 | 0.004 | 0.009 | 5.3E-03 | 3.2E-03 | 0.002 | 0.002 | 0.002 | 2.0E-03 | 0.0E+00 | 0.004 | 0.002 | 0.004 | 3.3E-03 | 1.2E-03 |
|  |  | *S. maltophilia* | 0.04 | 0.03 | 0.08 | 5.0E-02 | 2.6E-02 | 0.03 | 0.02 | 0.02 | 2.3E-02 | 5.8E-03 | 0.03 | 0.02 | 0.03 | 2.7E-02 | 5.8E-03 |
| 43 | Streptomyces | *S. albogriseolus* | 0.00002 | 0.00003 | 0.00001 | 2.0E-05 | 1.0E-05 | 0.0001 | 0.00003 | 0.00005 | 6.0E-05 | 3.6E-05 | 0.000003 | 0.00003 | 0 | 1.1E-05 | 1.7E-05 |
|  |  | *S. aureus* | 0.002 | 0.002 | 0.001 | 1.7E-03 | 5.8E-04 | 0.004 | 0.003 | 0.003 | 3.3E-03 | 5.8E-04 | 0.0007 | 0.002 | 0.0008 | 1.2E-03 | 7.2E-04 |
|  |  | *S. bikiniensis* | 0.0006 | 0.0005 | 0.0003 | 4.7E-04 | 1.5E-04 | 0.0009 | 0.0008 | 0.0008 | 8.3E-04 | 5.8E-05 | 0.0002 | 0.0004 | 0.0002 | 2.7E-04 | 1.2E-04 |
|  |  | *S. galbus* | 0.001 | 0.0008 | 0.0005 | 7.7E-04 | 2.5E-04 | 0.002 | 0.001 | 0.002 | 1.7E-03 | 5.8E-04 | 0.0002 | 0.0009 | 0.0003 | 4.7E-04 | 3.8E-04 |
| 44 | Variovorax | *V. boronicumulans* | 0.008 | 0.006 | 0.01 | 8.0E-03 | 2.0E-03 | 0.007 | 0.006 | 0.006 | 6.3E-03 | 5.8E-04 | 0.007 | 0.003 | 0.004 | 4.7E-03 | 2.1E-03 |
| 45 | Yersinia | *Y. frederiksenii* | 0.001 | 0.002 | 0.001 | 1.3E-03 | 5.8E-04 | 0.002 | 0.001 | 0.001 | 1.3E-03 | 5.8E-04 | 0.002 | 0.001 | 0.002 | 1.7E-03 | 5.8E-04 |

**Supplementary Table S2**. Relative abundance of bioremediation fungal species found in different locations of the river Ganga and Yamuna

| **Sl. No** | **Genus** | **Species** | **K1** | **K2** | **K3** | **Average** | **SD** | **F1** | **F2** | **F3** | **Average** | **SD** | **ND1** | **ND2** | **ND3** | **Average** | **SD** |
| --- | --- | --- | --- | --- | --- | --- | --- | --- | --- | --- | --- | --- | --- | --- | --- | --- | --- |
| 1 | Aspergillus | *A. flavus* | 0.050 | 0.030 | 0.060 | 4.7E-02 | 1.5E-02 | 0 | 0.05 | 0.05 | 3.3E-02 | 2.9E-02 | 0.03 | 0.04 | 0.07 | 4.7E-02 | 2.1E-02 |
|  |  | *A. nidulans* | 0.2 | 0.3 | 0.2 | 2.3E-01 | 5.8E-02 | 0 | 3 | 0.1 | 1.0E+00 | 1.7E+00 | 0.2 | 0.2 | 0.2 | 2.0E-01 | 3.4E-17 |
|  |  | *A. niger* | 0.2 | 0.3 | 0.3 | 2.7E-01 | 5.8E-02 | 0 | 0.2 | 0.1 | 1.0E-01 | 1.0E-01 | 0.2 | 0.2 | 0.2 | 2.0E-01 | 3.4E-17 |
|  |  | *A. oryzae* | 0.02 | 0.05 | 0.03 | 3.3E-02 | 1.5E-02 | 0 | 0.04 | 0.02 | 2.0E-02 | 2.0E-02 | 0.03 | 0.05 | 0.1 | 6.0E-02 | 3.6E-02 |
|  |  | *A. terreus* | 1 | 3 | 2 | 2.0E+00 | 1.0E+00 | 0 | 0.2 | 0.2 | 1.3E-01 | 1.2E-01 | 0.2 | 0.3 | 0.3 | 2.7E-01 | 5.8E-02 |
|  |  | *A. versicolor* | 0 | 0 | 0.003 | 1.0E-03 | 1.7E-03 | 0 | 0.002 | 0.0003 | 7.7E-04 | 1.1E-03 | 0.08 | 0.2 | 0.2 | 1.6E-01 | 6.9E-02 |
| 2 | Bjerkandera | *B. adusta* | 0 | 0 | 0 | 0.0E+00 | 0.0E+00 | 0 | 0.004 | 0 | 1.3E-03 | 2.3E-03 | 0 | 0 | 0 | 0.0E+00 | 0.0E+00 |
|  |  | *B. adusta* | 0 | 0 | 0 | 0.0E+00 | 0.0E+00 | 0 | 0.004 | 0 | 1.3E-03 | 2.3E-03 | 0 | 0 | 0 | 0.0E+00 | 0.0E+00 |
| 3 | Clonostachys | *C. rosea* | 0.004 | 0.002 | 0 | 2.0E-03 | 2.0E-03 | 0 | 0.002 | 0.0003 | 7.7E-04 | 1.1E-03 | 0.002 | 0.004 | 0 | 2.0E-03 | 2.0E-03 |
| 4 | Coprinus | *C. comatus* | 0 | 0 | 0 | 0.0E+00 | 0.0E+00 | 0 | 0 | 0.0003 | 1.0E-04 | 1.7E-04 | 0 | 0 | 0 | 0.0E+00 | 0.0E+00 |
| 5 | Exophiala | *E. xenobiotica* | 0.2 | 0.2 | 0.2 | 2.0E-01 | 3.4E-17 | 0 | 0.2 | 0.1 | 1.0E-01 | 1.0E-01 | 0.1 | 0.2 | 0.1 | 1.3E-01 | 5.8E-02 |
| 6 | Fusarium | *F. solani* | 0 | 0.002 | 0 | 6.7E-04 | 1.2E-03 | 0 | 0 | 0.007 | 2.3E-03 | 4.0E-03 | 0.2 | 0.3 | 0.3 | 2.7E-01 | 5.8E-02 |
| 7 | Mucor | *M. racemosus* | 0 | 0 | 0 | 0.0E+00 | 0.0E+00 | 0 | 0 | 0.001 | 3.3E-04 | 5.8E-04 | 0 | 0 | 0 | 0.0E+00 | 0.0E+00 |
| 8 | Penicillium | *P. crustosum* | 0 | 0 | 0 | 0.0E+00 | 0.0E+00 | 0 | 0 | 0.001 | 3.3E-04 | 5.8E-04 | 0 | 0 | 0.002 | 6.7E-04 | 1.2E-03 |
| 9 | Phanerochaete | *P.chrysosporium* | 0.01 | 0.005 | 0.01 | 8.3E-03 | 2.9E-03 | 0 | 0.007 | 0.003 | 3.3E-03 | 3.5E-03 | 0 | 0.006 | 0 | 2.0E-03 | 3.5E-03 |
|  |  | *P.chrysosporium* | 0.01 | 0.005 | 0.01 | 8.3E-03 | 2.9E-03 | 0 | 0.007 | 0.003 | 3.3E-03 | 3.5E-03 | 0 | 0.006 | 0 | 2.0E-03 | 3.5E-03 |
|  |  | *P. sordida* | 0 | 0 | 0 | 0.0E+00 | 0.0E+00 | 0 | 0 | 0 | 0.0E+00 | 0.0E+00 | 0 | 0 | 0.004 | 1.3E-03 | 2.3E-03 |
| 10 | Pleurotus | *P. eryngii* | 0.002 | 0 | 0 | 6.7E-04 | 1.2E-03 | 0 | 0 | 0 | 0.0E+00 | 0.0E+00 | 0 | 0 | 0 | 0.0E+00 | 0.0E+00 |
|  |  | *P. ostreatus* | 0.2 | 0.1 | 0.2 | 1.7E-01 | 5.8E-02 | 0 | 0.2 | 0.2 | 1.3E-01 | 1.2E-01 | 0.7 | 0.2 | 0.3 | 4.0E-01 | 2.6E-01 |
| 11 | Rhizopus | *R. oryzae* | 0 | 0.007 | 0 | 2.3E-03 | 4.0E-03 | 0 | 0 | 0.0003 | 1.0E-04 | 1.7E-04 | 0 | 0.001 | 0.004 | 1.7E-03 | 2.1E-03 |
|  |  | *R. stolonifer* | 0 | 0.002 | 0 | 6.7E-04 | 1.2E-03 | 0 | 0 | 0 | 0.0E+00 | 0.0E+00 | 0 | 0.001 | 0.002 | 1.0E-03 | 1.0E-03 |
| 12 | Trametes | *T. hirsuta* IBB 450 | 0 | 0 | 0 | 0.0E+00 | 0.0E+00 | 0 | 0 | 0 | 0.0E+00 | 0.0E+00 | 0 | 0 | 0.002 | 6.7E-04 | 1.2E-03 |
|  |  | *T. versicolor* | 0.3 | 0.2 | 0.2 | 2.3E-01 | 5.8E-02 | 0 | 0.2 | 0.1 | 1.0E-01 | 1.0E-01 | 0.04 | 0.08 | 0.07 | 6.3E-02 | 2.1E-02 |
| 13 | Trichoderma | *T. harzianum* | 0.2 | 0.2 | 0.2 | 2.0E-01 | 3.4E-17 | 0 | 0.2 | 0.1 | 1.0E-01 | 1.0E-01 | 0.03 | 0.08 | 0.05 | 5.3E-02 | 2.5E-02 |

**Supplementary Table S3**. Conserve domains related to bioremediation

| **Farakka Stretch** | | | | | | | | | |
| --- | --- | --- | --- | --- | --- | --- | --- | --- | --- |
| **Query** | | **E-Value** | | **Accession** | | **Short name** | | **Super family** | **Description** |
| >Scaffold_518_2_  Bacillus_cereus-F1_(+1) | | 7.72E-07 | | cd03297 | | ABC_ModC_molybdenum_transporter C | | cl25403 | ModC is an ABC-type transporter and the ATPase component of a molybdate transport system that also includes the periplasmic binding protein ModA and the membrane protein ModB. |
| >Scaffold_54988  _1_Rhodococcus_qingshengii-F1_(+1) | | 1.28E-22 | | cd03297 | | ABC_ModC_molybdenum_transporter | | cl25403 | ModC is an ABC-type transporter and the ATPase component of a molybdate transport system that also includes the periplasmic binding protein ModA and the membrane protein ModB. |
| >Scaffold_211618_  1_Acinetobacter_oleivorans-F1_(+1) | | 1.29E-17 | | TIGR02789 | | nickel_nikB | | cl28564 | This family consists of the NikB family of nickel ABC transporter permeases. |
| >Scaffold_54988_1_  Rhodococcus_qingshengii-F1_(+1) | | 2.26E-07 | | TIGR02770 | | nickel_nikD | | cl28181 | This family represents the NikD subunit of a multisubunit nickel import ABC transporter complex. |
| >Scaffold_25459  _1_Alcaligenes_faecalis-F1_(+1) | | 3.27E-08 | | TIGR03411 | | urea_trans_UrtD | | cl28181 | Members of this protein family are ABC transporter ATP-binding subunits associated with urea transport and metabolism. |
| >Scaffold_518_  2_Bacillus_cereus-1_(+1) | | 5.07E-06 | | TIGR03411 | | urea_trans_UrtD | | cl28181 | Members of this protein family are ABC transporter ATP-binding subunits associated with urea transport and metabolism. |
| >Scaffold_25459_1_  Alcaligenes_faecalis-F1_(+1) | | 0.0096299 | | TIGR03410 | | urea_trans_UrtE | | cl28181 | Members of this protein family are ABC transporter ATP-binding subunits associated with urea transport and metabolism. |
| >Scaffold_518_2_  Bacillus_cereus-F1_(+1) | | 2.06E-14 | | cd03225 | | ABC_cobalt_CbiO_domain1 | | cl25403 | Domain I of the ABC component of a cobalt transport family found in bacteria, archaea, and eukaryota. The transition metal cobalt is an essential component of many enzymes and must be transported into cells in appropriate amounts when needed. |
| >Scaffold_518_2_  Bacillus_cereus-F1_(+1) | | 1.50E-14 | | cd03226 | | ABC_cobalt_CbiO_domain2 | | cl25403 | Domain II of the ABC component of a cobalt transport family found in bacteria, archaea, and eukaryota. The transition metal cobalt is an essential component of many enzymes and must be transported into cells in appropriate amounts when needed. |
| >Scaffold_518_2_Bacillus_cereus-F1_(+1) | | 3.42E-12 | | cd03260 | | ABC_PstB_phosphate_transporter | | cl25403 | Phosphate uptake is of fundamental importance in the cell physiology of bacteria because phosphate is required as a nutrient. |
| >Scaffold_54988  _1_Rhodococcus_qingshengii-F1_(+1) | | 7.35E-15 | | cd03260 | | ABC_PstB_phosphate_transporter | | cl25403 | Phosphate uptake is of fundamental importance in the cell physiology of bacteria because phosphate is required as a nutrient. |
| >Scaffold_518_2_Bacillus_cereus-F1_(+1) | | 2.06E-14 | | cd03225 | | ABC_cobalt_CbiO_domain1 | | cl25403 | Domain I of the ABC component of a cobalt transport family found in bacteria, archaea, and eukaryota. The transition metal cobalt is an essential component of many enzymes and must be transported into cells in appropriate amounts when needed.. |
| >Scaffold_203100_1  _Bacillus_simplex-F1_(+1) | | 3.38E-13 | | cd03225 | | ABC_cobalt_CbiO_domain1 | | cl25403 | Domain I of the ABC component of a cobalt transport family found in bacteria, archaea, and eukaryota. The transition metal cobalt is an essential component of many enzymes and must be transported into cells in appropriate amounts when needed.. |
| >Scaffold_54988_1_Rhodococcus_qingshengii-F1_(+1) | | 7.91E-15 | | cd03225 | | ABC_cobalt_CbiO_domain1 | | cl25403 | Domain I of the ABC component of a cobalt transport family found in bacteria, archaea, and eukaryota. The transition metal cobalt is an essential component of many enzymes and must be transported into cells in appropriate amounts when needed. |
| >Scaffold_518_2  _Bacillus_cereus-F1_(+1) | | 0.000249125 | | PRK13647 | | cbiO | | cl28181 | This model describes the ATP binding subunit of the multisubunit cobalt transporter in bacteria and its equivalents in archaea. |
| >Scaffold_54988_1_Rhodococcus_qingshengii-F1_(+1) | | 2.81E-10 | | PRK13647 | | cbiO | | cl28181 | This model describes the ATP binding subunit of the multisubunit cobalt transporter in bacteria and its equivalents in archaea. |
| >Scaffold_54988_1_Rhodococcus_qingshengii-F1_(+1) | | 0.000911988 | | PRK10418 | | nikD | | cl28181 | This family represents the **NikD** subunit of a multisubunit nickel import ABC transporter complex. |
| >Scaffold_518_2_Bacillus_cereus-F1_(+1) | | 1.99E-11 | | PRK10419 | | nikE | | cl28181 | This family represents the **NikE** subunit of a multisubunit nickel import ABC transporter complex. |
| >Scaffold_54988_1_Rhodococcus_qingshengii-F1_(+1) | | 4.12E-09 | | PRK10419 | | nikE | | cl28181 | This family represents the **NikE** subunit of a multisubunit nickel import ABC transporter complex. |
| >Scaffold_211618_1_  Acinetobacter_oleivorans-F1_(+1) | | 1.29E-17 | | TIGR02789 | | nickel_nikB | | cl28564 | This family consists of the NikB family of nickel ABC transporter permeases. Operons that contain this protein also contain a homologous permease subunit NikC. Nickel is used in cells as part of urease or certain hydrogenases or superoxide dismutases. |
| >Scaffold_518_2_  Bacillus_cereus-F1_(+1) | | 5.00E-11 | | PRK11231 | | fecE | | cl28181 | iron-dicitrate transporter ATP-binding subunit |
| >Scaffold_54988_1_  Rhodococcus_qingshengii-F1_(+1) | | 6.06E-13 | | PRK11231 | | fecE | | cl28181 | iron-dicitrate transporter ATP-binding subunit |
| >Scaffold_518_2_Bacillus_cereus-F1_(+1) | | 2.29E-10 | | COG4619 | | FetA | | cl28181 | ABC-type iron transport system FetAB, ATPase component |
| >Scaffold_54988_1_Rhodococcus_qingshengii-F1_(+1) | | 1.89E-12 | | COG4619 | | FetA | | cl28181 | ABC-type iron transport system FetAB, ATPase component |
| >Scaffold_518_2_Bacillus_cereus-F1_(+1) | | 6.78E-10 | | PRK11432 | | fbpC | | cl28181 | ferric transporter ATP-binding subunit |
| >Scaffold_54988_1_Rhodococcus_qingshengii-F1_(+1) | | 1.04E-27 | | PRK11432 | | fbpC | | cl28181 | ferric transporter ATP-binding subunit |
| >Scaffold_7418_1_Desulfovibrio_desulfuricans-F1_(+1) | | 1.10E-05 | | COG4149 | | ModC | | cl00427 | ABC-type molybdate transport system, permease component |
| >Scaffold_518_2_Bacillus_cereus-F1_(+1) | | 5.76E-06 | | TIGR01978 | | sufC | | cl28181 | SufC is part of the SUF system, shown in E. coli to consist of six proteins and believed to act in Fe-S cluster formation during oxidative stress. |
| >Scaffold_518_2_Bacillus_cereus-F1_(+1) | | 2.33E-06 | | COG1117 | | PstB | | cl28181 | ABC-type phosphate transport system, ATPase component |
| >Scaffold_54988_1_Rhodococcus_qingshengii-F1_(+1) | | 7.17E-13 | | COG1117 | | PstB | | cl28181 | ABC-type phosphate transport system, ATPase component |
| Q#21 - >Scaffold_518_2_Bacillus_cereus-F1_(+1) | | 0.000132016 | | TIGR01184 | | ntrCD | | cl28181 | This model describes the ATP binding subunits of nitrate transport in bacteria and archaea. |
| >Scaffold_54988_1_Rhodococcus_qingshengii-F1_(+1) | | 2.59E-13 | | TIGR01184 | | ntrCD | | cl28181 | This model describes the ATP binding subunits of nitrate transport in bacteria and archaea. |
| >Scaffold_18593_1_Brevibacillus_borstelensis-F1_(+1) | | 7.71E-12 | | COG2205 | | KdpD | | cl27674 | This is a family of KdpD sensor kinase proteins that regulate the kdpFABC operon responsible for potassium transport. |
| >Scaffold_6572_2_Burkholderia_cenocepacia-F1_(+1) | | 8.14E-16 | | COG2205 | | KdpD | | cl27674 | This is a family of KdpD sensor kinase proteins that regulate the kdpFABC operon responsible for potassium transport. |
| >Scaffold_518_2_Bacillus_cereus-F1_(+1) | | 3.20E-17 | | cd03214 | | ABC_Iron-Siderophores_B12_Hemin | | cl25403 | ABC transporters, involved in the uptake of siderophores, heme, and vitamin B12, are widely conserved in bacteria and archaea. |
| >Scaffold_54988_1_Rhodococcus_qingshengii-F1_(+1) | | 1.78E-14 | | cd03214 | | ABC_Iron-Siderophores_B12_Hemin | | cl25403 | ABC transporters, involved in the uptake of siderophores, heme, and vitamin B12, are widely conserved in bacteria and archaea. |
| >Scaffold_518_2_Bacillus_cereus-F1_(+1) | | 0.00404196 | | PRK09984 | | PRK09984 | | cl28181 | phosphonate/organoposphate ester transporter subunit; |
| >Scaffold_54988_1_Rhodococcus_qingshengii-F1_(+1) | | 0.000772818 | | PRK09984 | | PRK09984 | | cl28181 | phosphonate/organophosphate ester transporter subunit. |
| >Scaffold_518_2_Bacillus_cereus-F1_(+1) | | 3.63E-08 | | TIGR01193 | | bacteriocin_ABC | | cl26602 | This model describes ABC-type bacteriocin transporter. |
| Q#127 - >Scaffold_54988_1_Rhodococcus_qingshengii-F1_(+1) | | 3.34E-05 | | TIGR01193 | | bacteriocin_ABC | | cl26602 | This model describes ABC-type bacteriocin transporter. |
| >Scaffold_52167_1_Burkholderia_xenovorans-F1_(+1) | | 1.61E-12 | | COG2124 | | CypX | | cl12078 | Cytochrome P450s are haem-thiolate proteins involved in the oxidative degradation of various compounds. They are particularly well known for their role in the degradation of environmental toxins and mutagens. |
| >Scaffold_5275_1_Rhizobium_meliloti-F1_(+1) | | 0.000469869 | | cd12808 | | Esterase_713_like-1 | | cl21494 | Uncharacterized enzymes similar to novel bacterial esterase that cleaves esters on halogenated cyclic compounds |
| >Scaffold_5275_1_Rhizobium_meliloti-F1_(+1) | | 0.000469869 | | cl21494 | | Abhydrolase superfamily | | cl21494 | A functionally diverse superfamily containing proteases, lipases, peroxidases, esterases, epoxide hydrolases and dehalogenases. |
| >Scaffold_518_2_Bacillus_cereus-F1_(+1) | | 0.00404196 | | PRK09984 | | PRK09984 | | cl28181 | phosphonate/organophosphate ester transporter subunit |
| >Scaffold_54988_1_Rhodococcus_qingshengii-F1_(+1) | | 0.000772818 | | PRK09984 | | PRK09984 | | cl28181 | phosphonate/organophosphate ester transporter subunit |
| >Scaffold_103791_1_Sphingobium_baderi-F1_(+1) | | 3.32E-11 | | cd01299 | | Met_dep_hydrolase_A | | cl00281 | Metallo-dependent hydrolases, subgroup A is part of the superfamily of metallo-dependent hydrolases. The vast majority of the members have a conserved metal binding site, involving four histidines and one aspartic acid residue. |
| >Scaffold_103791_1_Sphingobium_baderi-F1_(+1) | | 7.06E-08 | | cd01298 | | ATZ_TRZ_like | | cl00281 | TRZ/ATZ family contains enzymes from the atrazine degradation pathway and related hydrolases. |
| >Scaffold_103791_1_Sphingobium_baderi-F1_(+1) | | 1.17E-07 | | PRK09356 | | PRK09356 | | cl00281 | Superfamily of metallo-dependent hydrolases (also called amidohydrolase superfamily) is a large group of proteins that show conservation in their 3-dimensional fold (TIM barrel) and in details of their active site. The vast majority of the members have a conserved metal binding site, involving four histidines and one aspartic acid residue. |
| >Scaffold_103791_1_Sphingobium_baderi-F1_(+1) | | 3.91E-06 | | PRK06038 | | PRK06038 | | cl00281 | N-ethylammeline chlorohydrolase |
| >Scaffold_103791_1_Sphingobium_baderi-F1_(+1) | | 1.12E-06 | | COG0402 | | SsnA | | cl00281 | Superfamily of metallo-dependent hydrolases (also called amidohydrolase superfamily) is a large group of proteins that show conservation in their 3-dimensional fold (TIM barrel) and in details of their active site. The vast majority of the members have a conserved metal binding site, involving four histidines and one aspartic acid residue. |
| >Scaffold_103791_1_Sphingobium_baderi-F1_(+1) | | 4.91E-06 | | PRK05985 | | PRK05985 | | cl00281 | Superfamily of metallo-dependent hydrolases is a large group of proteins that show conservation in their 3-dimensional fold (TIM barrel) and in details of their active site. |
| **Kanpur Stretch** | | | | | | | | | |
| >NODE_537681_length_168_Ochrobactrum_anthropi-K1_(+1) | 0.001705 | | TIGR03407 | | urea_ABC_UrtA | | cl10011 | Members of this protein family are ABC transporter substrate-binding proteins associated with urea transport and metabolism. | |
| >NODE_1626103_length_165_Nitrobacter_hamburgensis-K1_(+1) | 6.91E-05 | | PRK11033 | | zntA | | cl27747 | zinc/cadmium/mercury/lead-transporting ATPase. | |
| >NODE_610119_length_249_Serratia_marcescens-K1_(+1) | 1.20E-24 | | cd03225 | | ABC_cobalt_CbiO_domain1 | | cl25403 | Domain I of the ABC component of a cobalt transport family found in bacteria, archaea, and eukaryota. | |
| >NODE_1835217_length_259_Yersinia_frederiksenii-K1_(+1) | 5.88E-07 | | cd08489 | | PBP2_NikA | | cl01709 | This family represents the periplasmic substrate-binding domain of nickel transport system, which functions in the import of nickel and in the control of chemotactic response away from nickel. | |
| >NODE_1626103_length_165_Nitrobacter_hamburgensis-K1_(+1) | 9.65E-07 | | cd07546 | | P-type_ATPase  _Pb_Zn_Cd2-like | | cl21460 | P-type heavy metal-transporting ATPase, similar to Escherichia coli ZntA which is selective for Pb(2+), Zn(2+), and Cd(2+)  Escherichia coli ZntA mediates resistance to toxic levels of selected divalent metal ions. ZntA has the highest selectivity for Pb(2+), followed by Zn(2+) and Cd(2+). | |
| >NODE_721102_length_176_Rhodobacter_sphaeroides-K1_(+1) | 0.007341 | | cd00625 | | ArsB_NhaD_permease | | cl21473 | nion permease ArsB/NhaD. These permeases have been shown to translocate sodium, arsenate, antimonite, sulfate and organic anions across biological membranes in all three kingdoms of life. | |
| >NODE_721102_length_176_Rhodobacter_sphaeroides-K1_(+1) | 1.84E-08 | | cl21473 | | ArsB_NhaD_permease superfamily | | - | Anion permease ArsB/NhaD. These permeases have been shown to translocate sodium, arsenate, antimonite, sulfate and organic anions across biological membranes in all three kingdoms of life. | |
| **Yamuna Stretch** | | | | | | | | | |
| >Gene_51972_Scaffold_11574_Cupriavidus_necator-Y1_(-1) | 0.003198 | | cl27511 | | Na_Ca_ex superfamily | | - | This family consist of the C proteins (C', C, Y1, Y2) found in Paramyxovirinae; human parainfluenza, and sendai virus. The C proteins effect viral RNA synthesis having both a positive and negative effect during the course of infection. | |
| >Gene_326272_Scaffold_209177_Sphingomonas_paucimobilis-Y1_(-1) | 0.002622 | | cl27511 | | Na_Ca_ex superfamily | |  | This family consist of the C proteins (C', C, Y1, Y2) found in Paramyxovirinae; human parainfluenza, and sendai virus. The C proteins effect viral RNA synthesis having both a positive and negative effect during the course of infection. | |
| >Gene_120604_Scaffold_41832_Achromobacter_xylosoxidans-Y1_(+1) | 0.000492 | | smart00327 | | VWA | | cl00057 | VWA domains in extracellular eukaryotic proteins mediate adhesion via metal ion-dependent adhesion sites (MIDAS). Intracellular VWA domains and homologues in prokaryotes have recently been identified. The proposed VWA domains in integrin beta subunits have recently been substantiated using sequence-based methods. | |
| >Gene_90290_Scaffold_26519_Comamonas_testosteroni-Y1_(+1) | 6.14E-07 | | cl25403 | | ABC_ATPase superfamily | | - | ABC transporters are a large family of proteins involved in the transport of a wide variety of different compounds, like sugars, ions, peptides, and more complex organic molecules. | |
| >Gene_224608_Scaffold_113506_Desulfovibrio_vulgaris-Y1_(+1) | 1.69E-60 | | cl25403 | | ABC_ATPase superfamily | | - | ABC transporters are a large family of proteins involved in the transport of a wide variety of different compounds, like sugars, ions, peptides, and more complex organic molecules. | |
| >Gene_13108_Scaffold_1744_Rhodoferax_ferrireducens-Y1_(+1) | 9.05E-99 | | cd08419 | | PBP2_CbbR_RubisCO_like | | cl25412 | The C-terminal substrate binding of LysR-type transcriptional regulator (CbbR) of RubisCO operon, which is involved in the carbon dioxide fixation, contains the type 2 periplasmic binding fold. | |
| >Gene_4373_Scaffold_553_Pseudomonas_fulva-Y1_(+1) | 1.63E-110 | | PRK09328 | | PRK09328 | | cl28093 | Superfamily of metallo-dependent hydrolases is a large group of proteins that show conservation in their 3-dimensional fold (TIM barrel) and in details of their active site. The vast majority of the members have a conserved metal binding site, involving four histidines and one aspartic acid residue. | |
| **Pesticide biodegradation** | | | | | | | | | |
| Q#91 - >Scaffold_52167_1_Burkholderia_xenovorans-F1_(+1) | 1.61E-12 | | cl12078 | | p450 superfamily | | - | Cytochrome P450s are haem-thiolate proteins involved in the oxidative degradation of various compounds. They are particularly well known for their role in the degradation of environmental toxins and mutagens. | |
| Q#77 - >Gene_214715_Scaffold_105530_Flavobacterium_aquatile-Y1_(+1) | 1.43E-77 | | cl25409 | | SDR superfamily | | - | SDRs are a functionally diverse family of oxidoreductases that have a single domain with a structurally conserved Rossmann fold (alpha/beta folding pattern with a central beta-sheet), an NAD(P)(H)-binding region, and a structurally diverse C-terminal region. | |
| Q#151 - >Gene_554991_Scaffold_501533_Sphingobium_quisquiliarum-Y1_(+1) | 5.03E-09 | | cl25409 | | SDR superfamily | | - | SDRs are a functionally diverse family of oxidoreductases that have a single domain with a structurally conserved Rossmann fold (alpha/beta folding pattern with a central beta-sheet), an NAD(P)(H)-binding region, and a structurally diverse C-terminal region. | |
| Q#15 - >NODE_260713_length_160_Alcanivorax_borkumensis-K1_(+1) | 2.43E-19 | | cl25409 | | SDR superfamily | | - | SDRs are a functionally diverse family of oxidoreductases that have a single domain with a structurally conserved Rossmann fold (alpha/beta folding pattern with a central beta-sheet), an NAD(P)(H)-binding region, and a structurally diverse C-terminal region. | |
| Q#121 - >NODE_1579811_length_152_Pseudomonas_veronii-K1_(+1) | 2.36E-13 | | cl25409 | | SDR superfamily | | - | SDRs are a functionally diverse family of oxidoreductases that have a single domain with a structurally conserved Rossmann fold (alpha/beta folding pattern with a central beta-sheet), an NAD(P)(H)-binding region, and a structurally diverse C-terminal region. | |
| Q#5 - >NODE_253856_length_178_Acinetobacter_calcoaceticus-K1_(+1) | 8.24E-27 | | cd04302 | | HAD_5NT | | cl21460 | 5'-nucleotidases dephosphorylate nucleoside 5'-monophosphates to nucleosides and inorganic phosphate. Purified Pseudomonas aeruginosa PA0065 displayed high activity toward 5'-UMP and 5'-IMP, significant activity against 5'-XMP and 5'-TMP, and low activity against 5'-CMP. | |
| Q#5 - >NODE_253856_length_178_Acinetobacter_calcoaceticus-K1_(+1) | 8.24E-27 | | cl21460 | | HAD_like superfamily | | - | The haloacid dehalogenase (HAD) superfamily includes carbon and phosphorus hydrolases such as 2-haloalkonoate dehalogenase, epoxide hydrolase, phosphoserine phosphatase, phosphomannomutase, phosphoglycolate phosphatase, P-type ATPase, among others. | |
| Q#83 - >NODE_1626103_length_165_Nitrobacter_hamburgensis-K1_(+1) | 7.13E-16 | | cl21460 | | HAD_like superfamily | | - | The haloacid dehalogenase (HAD) superfamily includes carbon and phosphorus hydrolases such as 2-haloalkonoate dehalogenase, epoxide hydrolase, phosphoserine phosphatase, phosphomannomutase, phosphoglycolate phosphatase, P-type ATPase, among others. | |
| Q#5 - >NODE_253856_length_178_Acinetobacter_calcoaceticus-K1_(+1) | 1.50E-10 | | cd01427 | | HAD_like | |  | The haloacid dehalogenase-like (HAD) superfamily includes L-2-haloacid dehalogenase, epoxide hydrolase, phosphoserine phosphatase, phosphomannomutase, phosphoglycolate phosphatase, P-type ATPase, and many others | |
| Q#5 - >NODE_253856_length_178_Acinetobacter_calcoaceticus-K1_(+1) | 2.77E-08 | | pfam00702 | | Hydrolase | | cl26185 | This family is structurally different from the alpha/beta hydrolase family (pfam00561). This family includes L-2-haloacid dehalogenase, epoxide hydrolases and phosphatases. | |
| Q#5 - >NODE_253856_length_178_Acinetobacter_calcoaceticus-K1_(+1) | 2.14E-06 | | TIGR01549 | | HAD-SF-IA-v1 | | cl21460 | The haloacid dehalogenase (HAD) superfamily includes carbon and phosphorus hydrolases | |
| Q#5 - >NODE_253856_length_178_Acinetobacter_calcoaceticus-K1_(+1) | 2.50E-06 | | cd07512 | | HAD_PGPase | | cl21460 | This family belongs to the haloacid dehalogenase-like (HAD) hydrolases, a large superfamily of diverse enzymes that catalyze carbon or phosphoryl group transfer reactions on a range of substrates, using an active site aspartate in nucleophilic catalysis. | |
| Q#5 - >NODE_253856_length_178_Acinetobacter_calcoaceticus-K1_(+1) | 2.35E-05 | | cd16417 | | HAD_PGPase | | cl21460 | Phosphoglycolate phosphatase (PGP; EC 3.1.3.18) catalyzes the conversion of 2-phosphoglycolate into glycolate and phosphate. Members of this family belong to the haloacid dehalogenase-like (HAD) hydrolases, a large superfamily of diverse enzymes that catalyze carbon or phosphoryl group transfer reactions on a range of substrates, using an active site aspartate in nucleophilic catalysis. | |
| Q#5 - >NODE_253856_length_178_Acinetobacter_calcoaceticus-K1_(+1) | 0.004985 | | pfam13419 | | HAD_2 | | cl26185 | - Haloacid dehalogenase-like hydrolase | |
| Q#51 - >NODE_451102_length_366_Comamonas_testosteroni-K1_(+1) | 8.10E-32 | | cd03479 | | Rieske_RO_Alpha_PhDO_like | | cl00938 | Rieske non-heme iron oxygenase (RO) family, Phthalate 4,5-dioxygenase (PhDO)-like subfamily, N-terminal Rieske domain of the oxygenase alpha subunit; composed of the oxygenase alpha subunits of PhDO and similar proteins including 3-chlorobenzoate 3,4-dioxygenase (CBDO), phenoxybenzoate dioxygenase (POB-dioxygenase) and 3-nitrobenzoate oxygenase (MnbA). ROs comprise a large class of aromatic ring-hydroxylating dioxygenases that enable microorganisms to tolerate and utilize aromatic compounds for growth | |
| Q#51 - >NODE_451102_length_366_Comamonas_testosteroni-K1_(+1) | 8.10E-32 | | cl00938 | | Rieske superfamily | | cl00938 | Rieske domain; a [2Fe-2S] cluster binding domain commonly found in Rieske non-heme iron oxygenase (RO) systems such as naphthalene and biphenyl dioxygenases, as well as in plant/cyanobacterial chloroplast b6f and mitochondrial cytochrome bc(1) complexes. | |
| Q#51 - >NODE_451102_length_366_Comamonas_testosteroni-K1_(+1) | 1.76E-11 | | cd03469 | | Rieske_RO_Alpha_N | | cl00938 | Rieske non-heme iron oxygenase (RO) family, N-terminal Rieske domain of the oxygenase alpha subunit; The RO family comprise a large class of aromatic ring-hydroxylating dioxygenases found predominantly in microorganisms. These enzymes enable microorganisms to tolerate and even exclusively utilize aromatic compounds for growth. | |
| Q#51 - >NODE_451102_length_366_Comamonas_testosteroni-K1_(+1) | 5.64E-11 | | cd03548 | | Rieske_RO_Alpha_OMO_CARDO | | cl00938 | Rieske non-heme iron oxygenase (RO) family, N-terminal Rieske domain of the oxygenase alpha subunit; The RO family comprise a large class of aromatic ring-hydroxylating dioxygenases found predominantly in microorganisms. These enzymes enable microorganisms to tolerate and even exclusively utilize aromatic compounds for growth. | |
| Q#51 - >NODE_451102_length_366_Comamonas_testosteroni-K1_(+1) | 3.20E-10 | | cl28556 | | HcaE superfamily | | cl34797 | Phenylpropionate dioxygenase or related ring-hydroxylating dioxygenase, large terminal subunit | |
| Q#153 - >NODE_1344277_length_154_Streptomyces_galbus-K1_(+1) | 0.001597 | | cl28556 | | HcaE superfamily | | cl34797 | - Phenylpropionate dioxygenase or related ring-hydroxylating dioxygenase, large terminal subunit | |

**Supplementary Table S4. Evidence of pollutant degrading bacteria mentioned in present study**

| **Sl. No** | **Genus** | **Name of species** | **Target pollutant** | **Reference paper** |
| --- | --- | --- | --- | --- |
| 1 | Achromobacter | *A. xylosoxidans* | Phenoxyacetic acid | Vedler, E., Kõiv, V., & Heinaru, A. (2000). Analysis of the 2, 4-dichlorophenoxyacetic acid-degradative plasmid pEST4011 of Achromobacter xylosoxidans subsp. denitrificans strain EST4002. Gene, 255(2), 281-288. |
| 2 | Acinetobacter | *A. baumannii* | Deltamethrin | Zhan, H., Wang, H., Liao, L., Feng, Y., Fan, X., Zhang, L., & Chen, S. (2018). Kinetics and novel degradation pathway of permethrin in Acinetobacter baumannii ZH-14. *Frontiers in microbiology*, *9*, 98. |
|  |  | *A. calcoaceticus* | Chlorpyrifos | Zhao, L., Wang, F., & Zhao, J. (2014). Identification and functional characteristics of chlorpyrifos‐degrading and plant growth promoting bacterium Acinetobacter calcoaceticus. *Journal of basic microbiology*, *54*(5), 457-463.  Akbar, S., & Sultan, S. (2016). Soil bacteria showing a potential of chlorpyrifos degradation and plant growth enhancement. *brazilian journal of microbiology*, *47*(3), 563-570. |
|  |  | *A. oleivorans* | Fipronil | Uniyal, S., Paliwal, R., Verma, M., Sharma, R. K., & Rai, J. P. N. (2016). Isolation and characterization of fipronil degrading Acinetobacter calcoaceticus and Acinetobacter oleivorans from rhizospheric zone of Zea mays. Bulletin of environmental contamination and toxicology, 96(6), 833-838. |
| 3 | Aeromonas | *A. hydrophila* | Phenoxyacetic acid | Markusheva, T.V., Zhurenko, E.Y., Galkin, E.G., Korobov, V.V., Zharikova, N.V. and Gafiyatova, L.R., 2004. Identification and characterization of a plasmid in strain Aeronomas hydrophila IBRB-36 4CPA carrying genes for catabolism of chlorophenoxyacetic acids. *Russian Journal of Genetics*, *40*(11), pp.1210-1214. |
|  |  | *A. salmonicida* | Benzo[a]pyrene | Aziz, A., Agamuthu, P., Alaribe, F.O. and Fauziah, S.H., 2018. Biodegradation of benzo [a] pyrene by bacterial consortium isolated from mangrove sediment. *Environmental technology*, *39*(4), pp.527-535. |
|  |  | *A. sobria* | Permethrin | Lee, S., Gan, J., Kim, J.S., Kabashima, J.N. and Crowley, D.E., 2004. Microbial transformation of pyrethroid insecticides in aqueous and sediment phases. Environmental Toxicology and Chemistry: An International Journal, 23(1), pp.1-6. |
| 4 | Alcaligenes | *A. faecalis* | PAHs | Andreolli, M., Lampis, S., Zenaro, E., Salkinoja-Salonen, M. and Vallini, G., 2011. Burkholderia fungorum DBT1: a promising bacterial strain for bioremediation of PAHs-contaminated soils. *FEMS microbiology letters*, *319*(1), pp.11-18. |
| 5 | Alcanivorax | *A. borkumensis* | Crude oil | Santisi, S., Cappello, S., Catalfamo, M., Mancini, G., Hassanshahian, M., Genovese, L., … Yakimov, M. M. (2015). Biodegradation of crude oil by individual bacterial strains and a mixed bacterial consortium. *Brazilian journal of microbiology: [publication of the Brazilian Society for Microbiology]*, *46*(2), 377–387. doi:10.1590/S1517-838246120131276. |
| 6 | Azotobacter | *A. chroococcum* | Phenoxyacetic acid | Balajee, S. and Mahadevan, A., 1990. Dissimilation of 2, 4-dichlorophenoxyacetic acid by Azotobacter chroococcum. *Xenobiotica*, *20*(6), pp.607-617. |
| 7 | Bacillus | *B. aerophilus* | Phorate | Jariyal, M., Jindal, V., Mandal, K., Gupta, V.K. and Singh, B., 2018. Bioremediation of organophosphorus pesticide phorate in soil by microbial consortia. *Ecotoxicology and environmental safety*, *159*, pp.310-316. |
|  |  | *B. cereus* | Glyphosate | Acosta-Cortés, A.G., Martinez-Ledezma, C., López-Chuken, U.J., Kaushik, G., Nimesh, S. and Villarreal-Chiu, J.F., 2019. Polyphosphate recovery by a native Bacillus cereus strain as a direct effect of glyphosate uptake. *The ISME journal*, *13*(6), pp.1497-1505. |
|  |  | *B. flexus* | Fenvalerate | Mulla, S.I., Ameen, F., Tallur, P.N., Bharagava, R.N., Bangeppagari, M., Eqani, S.A.M.A.S., Bagewadi, Z.K., Mahadevan, G.D., Yu, C.P. and Ninnekar, H.Z., 2017. Aerobic degradation of fenvalerate by a Gram-positive bacterium, Bacillus flexus strain XJU-4. *3 Biotech*, *7*(5), p.320. |
|  |  | *B. megaterium* | Quiclorac | Liu, M., Luo, K., Wang, Y., Zeng, A., Zhou, X., Luo, F. and Bai, L., 2014. Isolation, identification and characteristics of an endophytic quinclorac degrading bacterium Bacillus megaterium Q3. *PloS one*, *9*(9), p.e108012. |
|  |  | *B. niacini* | Metolachlor | Wang, Y.S., Liu, J.C., Chen, W.C. and Yen, J.H., 2008. Characterization of acetanilide herbicides degrading bacteria isolated from tea garden soil. *Microbial ecology*, *55*(3), pp.435-443. |
|  |  | *B. pseudomycoides* | Metolachlor | Wang, Y.S., Liu, J.C., Chen, W.C. and Yen, J.H., 2008. Characterization of acetanilide herbicides degrading bacteria isolated from tea garden soil. *Microbial ecology*, *55*(3), pp.435-443. |
|  |  | *B. safensis* | Chlorpyrifos, Malathion and Dimethoate | Ishag, A.E.S., Abdelbagi, A.O., Hammad, A.M., Elsheikh, E.A., Elsaid, O.E., Hur, J.H. and Laing, M.D., 2016. Biodegradation of chlorpyrifos, malathion, and dimethoate by three strains of bacteria isolated from pesticide-polluted soils in Sudan. *Journal of agricultural and food chemistry*, *64*(45), pp.8491-8498. |
|  |  | *B.simplex* | Metolachlor | Munoz, A., Koskinen, W.C., Cox, L. and Sadowsky, M.J., 2010. Biodegradation and mineralization of metolachlor and alachlor by Candida xestobii. *Journal of agricultural and food chemistry*, *59*(2), pp.619-627. |
|  |  | *B. subtilis* | Pendimethalin | Ni, H.Y., Wang, F., Li, N., Yao, L., Dai, C., He, Q., He, J. and Hong, Q., 2016. Pendimethalin nitroreductase is responsible for the initial pendimethalin degradation step in Bacillus subtilis Y3. *Appl. Environ. Microbiol.*, *82*(24), pp.7052-7062. |
|  |  | *B. tequilensis* | Trichlorfen | Tian, J., Yu, C., Xue, Y., Zhao, R., Wang, J. and Chen, L., 2016. Performance of trichlorfon degradation by a novel Bacillus tequilensis strain PA F-3 and its proposed biodegradation pathway. *Biodegradation*, *27*(4-6), pp.265-276. |
|  |  | *B. thuringiensis* | Metolachlor | Wang, Y.S., Liu, J.C., Chen, W.C. and Yen, J.H., 2008. Characterization of acetanilide herbicides degrading bacteria isolated from tea garden soil. *Microbial ecology*, *55*(3), pp.435-443. |
|  |  | *B. lentus* | Azo dye | Shanmugam, B.K., Easwaran, S.N., Lakra, R., Deepa, P.R. and Mahadevan, S., 2017. Metabolic pathway and role of individual species in the bacterial consortium for biodegradation of azo dye: a biocalorimetric investigation. *Chemosphere*, *188*, pp.81-89. |
| 8 | Bordetella | *B. petrii* | Endosulfan | Odukkathil, G. and Vasudevan, N., 2015. Biodegradation of endosulfan isomers and its metabolite endosulfate by two biosurfactant producing bacterial strains of Bordetella petrii. *Journal of Environmental Science and Health, Part B*, *50*(2), pp.81-89. |
| 9 | Brevibacillus | *B. borstelensis* | Carbendazim | Arya, R. and K Sharma, A., 2016. Bioremediation of carbendazim, a benzimidazole fungicide using Brevibacillus borstelensis and Streptomyces albogriseolus together. *Current pharmaceutical biotechnology*, *17*(2), pp.185-189. |
|  |  | *B. parabrevis* | Cypermethrin | Tang, J., Liu, B., Chen, T.T., Yao, K., Zeng, L., Zeng, C.Y. and Zhang, Q., 2018. Screening of a beta-cypermethrin-degrading bacterial strain Brevibacillus parabrevis BCP-09 and its biochemical degradation pathway. *Biodegradation*, *29*(6), pp.525-541. |
| 10 | Burkholderia | *B. cenocepacia* | Methyl parathion | Fernández‐López, M.G., Popoca‐Ursino, C., Sánchez‐Salinas, E., Tinoco‐Valencia, R., Folch‐Mallol, J.L., Dantán‐González, E. and Laura Ortiz‐Hernández, M., 2017. Enhancing methyl parathion degradation by the immobilization of Burkholderia sp. isolated from agricultural soils. *MicrobiologyOpen*, *6*(5), p.e00507. |
|  |  | *B. cepacia* | Phenoxyacetic acid | Ghadi, S.C. and Sangodkar, U.M., 1994. Identification of a meta-cleavage pathway for metabolism of phenoxyacetic acid and phenol in Pseudomonas cepacia AC1100. *Biochemical and biophysical research communications*, *204*(2), pp.983-993. |
|  |  | *B. vietnamiensis* | Trichloro ethylene | Hamid, S., Bae, W., Kim, S., & Amin, M. T. (2014). Enhancing co-metabolic degradation of trichloroethylene with toluene using Burkholderia vietnamiensis G4 encapsulated in polyethylene glycol polymer. *Environmental technology*, *35*(12), 1470-1477. |
|  |  | *B. gladioli* | Profenophos | Malghani, S., Chatterjee, N., Yu, H.X. and Luo, Z., 2009. Isolation and identification of profenofos degrading bacteria. *Brazilian Journal of Microbiology*, *40*(4), pp.893-900. |
| 11 | Chryseobacterium | *C. joostei* | Organochlorine pesticide | Krishna, K. R., & Philip, L. (2008). Biodegradation of mixed pesticides by mixed pesticide enriched cultures. *Journal of Environmental Science and Health, Part B*, *44*(1), 18-30. |
| 12 | Clostridium | *C. acetobutylicum* | 2,4,6-Trinitrotoluene and Other Nitroaromatic Compounds | Watrous, M. M., Clark, S., Kutty, R., Huang, S., Rudolph, F. B., Hughes, J. B., & Bennett, G. N. (2003). 2, 4, 6-Trinitrotoluene reduction by an Fe-only hydrogenase in Clostridium acetobutylicum. *Appl. Environ. Microbiol.*, *69*(3), 1542-1547. |
| 13 | Comamonas | *C. testosteroni* | Phenoxyacetic acid | Loffhagen, N., Härtig, C. and Babel, W., 2003. Energization of Comamonas testosteroni ATCC 17454 for indicating toxic effects of chlorophenoxy herbicides. *Archives of environmental contamination and toxicology*, *45*(3), pp.317-323. |
| 14 | Cupriavidus | *C. necator* | Phenoxyacetic acid | Ledger, T., Pieper, D.H. and González, B., 2006. Chlorophenol hydroxylases encoded by plasmid pJP4 differentially contribute to chlorophenoxyacetic acid degradation. *Appl. Environ. Microbiol.*, *72*(4), pp.2783-2792. |
|  |  | *C. taiwanensis* | Phenanthrene | Oyehan, T.A. and Al-Thukair, A.A., 2017. Isolation and characterization of PAH-degrading bacteria from the Eastern Province, Saudi Arabia. *Marine pollution bulletin*, *115*(1-2), pp.39-46. |
| 15 | Dechloromonas | *D. aromatica* | Perchlorate and oxidize chlorobenzoate, toluene, and xylene | Salinero, K.K., Keller, K., Feil, W.S., Feil, H., Trong, S., Di Bartolo, G. and Lapidus, A., 2009. Metabolic analysis of the soil microbe Dechloromonas aromatica str. RCB: indications of a surprisingly complex life-style and cryptic anaerobic pathways for aromatic degradation. *BMC genomics*, *10*(1), p.351. |
| 16 | Deinococcus | *D. radiodurans* | Di-n-butyl phthalate (DBP) | Liao, C.S., Chen, L.C., Chen, B.S. and Lin, S.H., 2010. Bioremediation of endocrine disruptor di-n-butyl phthalate ester by Deinococcus radiodurans and Pseudomonas stutzeri. *Chemosphere*, *78*(3), pp.342-346. |
| 17 | Desulfitobacterium | *D. hafniense* | Tetrachloroethene | Duret, A., Holliger, C. and Maillard, J., 2012. The physiological opportunism of Desulfitobacterium hafniense strain TCE1 towards organohalide respiration with tetrachloroethene. *Appl. Environ. Microbiol.*, *78*(17), pp.6121-6127. |
|  |  | *D. hafniense Y51* | Tetrachloroethene | Marozava, S., Vargas‐López, R., Tian, Y., Merl‐Pham, J., Braster, M., Meckenstock, R.U., Smidt, H., Röling, W.F. and Westerhoff, H.V., 2018. Metabolic flexibility of a prospective bioremediator: Desulfitobacterium hafniense Y51 challenged in chemostats. *Environmental microbiology*, *20*(7), pp.2652-2669. |
| 18 | Desulfovibrio | *D. desulfuricans* | Radionuclide | Barton, L.L., Lyle, D.A., Ritz, N.L., Granat, A.S., Khurshid, A.N., Kherbik, N., Hider, R. and Lin, H.C., 2016. Bismuth (III) deferiprone effectively inhibits growth of Desulfovibrio desulfuricans ATCC 27774. *BioMetals*, *29*(2), pp.311-319. |
|  |  | *D. vulgaris* | Cr(VI) | Franco, L.C., Steinbeisser, S., Zane, G.M., Wall, J.D. and Fields, M.W., 2018. Cr (VI) reduction and physiological toxicity are impacted by resource ratio in Desulfovibrio vulgaris. *Applied microbiology and biotechnology*, *102*(6), pp.2839-2850. |
| 19 | Enterobacter | *E. asburiae* | Acephate | Ramya, S.L., Venkatesan, T., Murthy, K.S., Jalali, S.K. and Varghese, A., 2016. Degradation of acephate by Enterobacter asburiae, Bacillus cereus and Pantoea agglomerans isolated from diamondback moth Plutella xylostella (L), a pest of cruciferous crops. *Journal of environmental biology*, *37*(4), p.611. |
|  |  | *E. cloacae* | Chlorpyrifos | Shabbir, M., Singh, M., Maiti, S., Kumar, S. and Saha, S.K., 2018. Removal enactment of organo-phosphorous pesticide using bacteria isolated from domestic sewage. *Bioresource technology*, *263*, pp.280-288. |
| 20 | Escherichia | *E. coli* | Malathion | Zhang, J., Lan, W., Qiao, C. and Jiang, H., 2004. Bioremediation of organophosphorus pesticides by surface‐expressed carboxylesterase from mosquito on Escherichia coli. *Biotechnology progress*, *20*(5), pp.1567-1571. |
| 21 | Flavobacterium | *F. aquatile* | Phenoxyacetic acid | Jensen, H. L. and Petersen H. I., 1952 Detoxification of hormone herbicide soil bacteria. Nature, 170, 39 |
| 22 | Geobacter | *G. metallireducens* | U(VI) | Icopini, G.A., Lack, J.G., Hersman, L.E., Neu, M.P. and Boukhalfa, H., 2009. Plutonium (V/VI) reduction by the metal-reducing bacteria Geobacter metallireducens GS-15 and Shewanella oneidensis MR-1. *Appl. Environ. Microbiol.*, *75*(11), pp.3641-3647. |
|  |  | *G. sulfurreducens* | U(VI) | Orellana, R., Hixson, K.K., Murphy, S., Mester, T., Sharma, M.L., Lipton, M.S. and Lovley, D.R., 2014. Proteome of Geobacter sulfurreducens in the presence of U (VI). *Microbiology*, *160*(12), pp.2607-2617. |
| 23 | Methylibium | *M. petroleiphilum* | Aromatic (benzene, toluene, and xylene) and straight-chain (C5 to C12) hydrocarbons | Kane, S.R., Chakicherla, A.Y., Chain, P.S., Schmidt, R., Shin, M.W., Legler, T.C., Scow, K.M., Larimer, F.W., Lucas, S.M., Richardson, P.M. and Hristova, K.R., 2007. Whole-genome analysis of the methyl tert-butyl ether-degrading beta-proteobacterium Methylibium petroleiphilum PM1. *Journal of bacteriology*, *189*(5), pp.1931-1945. |
| 24 | Nitrobacter | *N. hamburgensis* | Ammonium ion | Franco-Rivera, A., Paniagua-Michel, J. and Zamora-Castro, J., 2007. Characterization and performance of constructed nitrifying biofilms during nitrogen bioremediation of a wastewater effluent. *Journal of industrial microbiology & biotechnology*, *34*(4), pp.279-287. |
| 26 | Nitrosomonas | *N. europaea* | Ammonium ion | Stein, L.Y. and Arp, D.J., 1998. Loss of ammonia monooxygenase activity in Nitrosomonas europaea upon exposure to nitrite. *Appl. Environ. Microbiol.*, *64*(10), pp.4098-4102. |
| 27 | Novosphingobium | *N. pentaromativorans* | Polycyclic aromatic hydrocarbon | Lyu, Y., Zheng, W., Zheng, T. and Tian, Y., 2014. Biodegradation of polycyclic aromatic hydrocarbons by Novosphingobium pentaromativorans US6-1. *PLoS One*, *9*(7), p.e101438. |
| 28 | Ochrobactrum | *O. anthropi* | Benzo[a]pyrene | Aziz, A., Agamuthu, P., Alaribe, F.O. and Fauziah, S.H., 2018. Biodegradation of benzo [a] pyrene by bacterial consortium isolated from mangrove sediment. *Environmental technology*, *39*(4), pp.527-535. |
|  |  | *O. intermedium* | Phenanthrene | Oyehan, T.A. and Al-Thukair, A.A., 2017. Isolation and characterization of PAH-degrading bacteria from the Eastern Province, Saudi Arabia. *Marine pollution bulletin*, *115*(1-2), pp.39-46. |
| 29 | Pantoea | *P. agglomerans* | Acephate | Ramya, S.L., Venkatesan, T., Murthy, K.S., Jalali, S.K. and Varghese, A., 2016. Degradation of acephate by Enterobacter asburiae, Bacillus cereus and Pantoea agglomerans isolated from diamondback moth Plutella xylostella (L), a pest of cruciferous crops. *Journal of environmental biology*, *37*(4), p.611. |
| 30 | Paraburkholderia | *P. fungorum* | Polycyclic aromatic hydrocarbon | Khoei, N.S., Andreolli, M., Lampis, S., Vallini, G. and Turner, R.J., 2016. A comparison of the response of two Burkholderia fungorum strains grown as planktonic cells versus biofilm to dibenzothiophene and select polycyclic aromatic hydrocarbons. *Canadian journal of microbiology*, *62*(10), pp.851-860. |
|  |  | *P. xenovorans* | Carbamazepine | Aukema, K.G., Escalante, D.E., Maltby, M.M., Bera, A.K., Aksan, A. and Wackett, L.P., 2016. In silico identification of bioremediation potential: carbamazepine and other recalcitrant personal care products. *Environmental science & technology*, *51*(2), pp.880-888. |
| 31 | Paracoccus | *P. denitrificans* | N,N-Dimethylformamide | Zheng, Y., Chen, D., Li, N., Xu, Q., Li, H., He, J. and Lu, J., 2016. Efficient simultaneous adsorption-biodegradation of high-concentrated N, N-dimethylformamide from water by Paracoccus denitrificans-graphene oxide microcomposites. *Scientific reports*, *6*, p.20003. |
| 32 | Pseudomonas | *P. aeruginosa* | Cr(VI) | Tang, S., Yin, H., Zhou, S., Chen, S., Peng, H., Liu, Z. and Dang, Z., 2016. Simultaneous Cr (VI) removal and 2, 2′, 4, 4′-tetrabromodiphenyl ether (BDE-47) biodegradation by Pseudomonas aeruginosa in liquid medium. *Chemosphere*, *150*, pp.24-32. |
|  |  | *P. alcaligenes* | Phenoxyacetic acid | Harker, A.R., Olsen, R.H. and Seidler, R.J., 1989. Phenoxyacetic acid degradation by the 2, 4-dichlorophenoxyacetic acid (TFD) pathway of plasmid pJP4: mapping and characterization of the TFD regulatory gene, tfdR. *Journal of bacteriology*, *171*(1), pp.314-320. |
|  |  | *P. azotoformans* | Cyhalofop Butyl | Nie, Z.J., Hang, B.J., Cai, S., Xie, X.T., He, J. and Li, S.P., 2011. Degradation of cyhalofop-butyl (CyB) by Pseudomonas azotoformans strain QDZ-1 and cloning of a novel gene encoding CyB-hydrolyzing esterase. *Journal of agricultural and food chemistry*, *59*(11), pp.6040-6046. |
|  |  | *P. citronellolis* | Phenanthrene | Oyehan, T.A. and Al-Thukair, A.A., 2017. Isolation and characterization of PAH-degrading bacteria from the Eastern Province, Saudi Arabia. *Marine pollution bulletin*, *115*(1-2), pp.39-46.  Prakash, O., Kumari, K. and Lal, R., 2007. Pseudomonas delhiensis sp. nov., from a fly ash dumping site of a thermal power plant. *International journal of systematic and evolutionary microbiology*, *57*(3), pp.527-531. |
|  |  | *P. fluorescens* | Metolachlor | Zablotowicz, R.M., Locke, M.A., Hoagland, R.E., Knight, S.S. and Cash, B., 2001. Fluorescent Pseudomonas isolates from Mississippi Delta oxbow lakes: in vitro herbicide biotransformations. *Environmental Toxicology: An International Journal*, *16*(1), pp.9-19. |
|  |  | *P. fulva* | Alpha-cypermethrin | Yang, J., Feng, Y., Zhan, H., Liu, J., Yang, F., Zhang, K., Zhang, L. and Chen, S., 2018. Characterization of a pyrethroid-degrading Pseudomonas fulva strain P31 and biochemical degradation pathway of D-phenothrin. *Frontiers in microbiology*, *9*, p.1003. |
|  |  | *P. mendocina* | Cr(VI) | Viti, C., Decorosi, F., Tatti, E. and Giovannetti, L., 2007. Characterization of chromate‐resistant and‐reducing bacteria by traditional means and by a high‐throughput phenomic technique for bioremediation purposes. *Biotechnology progress*, *23*(3), pp.553-559. |
|  |  | *P. plecoglossicida* | Profenofos | Siripattanakul-Ratpukdi, S., Vangnai, A.S., Sangthean, P. and Singkibut, S., 2015. Profenofos insecticide degradation by novel microbial consortium and isolates enriched from contaminated chili farm soil. *Environmental Science and Pollution Research*, *22*(1), pp.320-328. |
|  |  | *P. pseudoalcaligenes* | Polychlorinated Biphenyls | Suenaga, H., Nonaka, K., Fujihara, H., Goto, M. and Furukawa, K., 2010. Hybrid pseudomonads engineered by two-step homologous recombination acquire novel degradation abilities toward aromatics and polychlorinated biphenyls. *Applied microbiology and biotechnology*, *88*(4), pp.915-923. |
|  |  | *P. putida* | Carbofuran | Gong, T., Xu, X., Dang, Y., Kong, A., Wu, Y., Liang, P., Wang, S., Yu, H., Xu, P. and Yang, C., 2018. An engineered Pseudomonas putida can simultaneously degrade organophosphates, pyrethroids and carbamates. *Science of The Total Environment*, *628*, pp.1258-1265. |
|  |  | *P. resinovorans* | Carbazole and 2,3-dichlorodibenzo-p-dioxin | Widada, J., Nojiri, H., Yoshida, T., Habe, H. and Omori, T., 2002. Enhanced degradation of carbazole and 2, 3-dichlorodibenzo-p-dioxin in soils by Pseudomonas resinovorans strain CA10. *Chemosphere*, *49*(5), pp.485-491. |
|  |  | *P. stutzeri* | di-n-butyl phthalate | Liao, C.S., Chen, L.C., Chen, B.S. and Lin, S.H., 2010. Bioremediation of endocrine disruptor di-n-butyl phthalate ester by Deinococcus radiodurans and Pseudomonas stutzeri. *Chemosphere*, *78*(3), pp.342-346. |
|  |  | *P. veronii* | Cr(VI) | Garavaglia, L., Cerdeira, S.B. and Vullo, D.L., 2010. Chromium (VI) biotransformation by β-and γ-Proteobacteria from natural polluted environments: a combined biological and chemical treatment for industrial wastes. *Journal of hazardous materials*, *175*(1-3), pp.104-110. |
| 33 | Pseudoxanthomonas | *P. suwonensis* | Profenofos | Talwar, M.P. and Ninnekar, H.Z., 2015. Biodegradation of pesticide profenofos by the free and immobilized cells of Pseudoxanthomonas suwonensis strain HNM. *Journal of basic microbiology*, *55*(9), pp.1094-1103. |
| 34 | Sinorhizobium | *R. meliloti* | Dibenzothiophene | Frassinetti, S., Setti, L., Corti, A., Farrinelli, P., Montevecchi, P. and Vallini, G., 1998. Biodegradation of dibenzothiophene by a nodulating isolate of Rhizobium meliloti. *Canadian journal of microbiology*, *44*(3), pp.289-297. |
| 35 | Rhodobacter | *R. sphaeroides* | Dichlorvos | Zhao, K., Yu, Y., Jiang, D., Wang, D., Li, Z.M., Huang, G.Z. and Bai, Z.H., 2009. Degradation of dichlorvos by Rhodobacter sphaeroides. *Huan jing ke xue= Huanjing kexue*, *30*(4), pp.1199-1204. |
| 36 | Rhodococcus | *R. erythropolis* | Carbendazim | Wang, Z., Xu, J., Li, Y., Wang, K., Wang, Y., Hong, Q., Li, W.J. and Li, S.P., 2010. Rhodococcus jialingiae sp. nov., an actinobacterium isolated from sludge of a carbendazim wastewater treatment facility. *International journal of systematic and evolutionary microbiology*, *60*(2), pp.378-381.  Xu, J.L., He, J., Wang, Z.C., Wang, K., Li, W.J., Tang, S.K. and Li, S.P., 2007. Rhodococcus qingshengii sp. nov., a carbendazim-degrading bacterium. *International journal of systematic and evolutionary microbiology*, *57*(12), pp.2754-2757. |
|  |  | *R. koreensis* | Endosulfan | Ito, K., Kawashima, F., Takagi, K., Kataoka, R., Kotake, M., Kiyota, H., Yamazaki, K., Sakakibara, F. and Okada, S., 2016. Isolation of endosulfan sulfate-degrading Rhodococcus koreensis strain S1-1 from endosulfan contaminated soil and identification of a novel metabolite, endosulfan diol monosulfate. *Biochemical and biophysical research communications*, *473*(4), pp.1094-1099. |
|  |  | *R. qingshengii* | Buprofezin | Chen, X., Ji, J., Zhao, L., Qiu, J., Dai, C., Wang, W., He, J., Jiang, J., Hong, Q. and Yan, X., 2017. Molecular mechanism and genetic determinants of buprofezin degradation. *Appl. Environ. Microbiol.*, *83*(18), pp.e00868-17. |
|  |  | *R. ruber* | Clodinafop propargyl | Hongming, L., Xu, L., Zhaojian, G., Fan, Y., Dingbin, C., Jianchun, Z., Jianhong, X., Shunpeng, L. and Qing, H., 2015. Isolation of an aryloxyphenoxy propanoate (AOPP) herbicide-degrading strain Rhodococcus ruber JPL-2 and the cloning of a novel carboxylesterase gene (feh). *Brazilian Journal of Microbiology*, *46*(2), pp.425-432. |
| 37 | Rhodoferax | *R. ferrireducens* | Fe(III) | Risso, C., Sun, J., Zhuang, K., Mahadevan, R., DeBoy, R., Ismail, W., Shrivastava, S., Huot, H., Kothari, S., Daugherty, S. and Bui, O., 2009. Genome-scale comparison and constraint-based metabolic reconstruction of the facultative anaerobic Fe (III)-reducer Rhodoferax ferrireducens. *BMC genomics*, *10*(1), p.447. |
| 38 | Serratia | *S. marcescens* | Metsulfuron Methyl | Zhang, H., Mu, W., Hou, Z., Wu, X., Zhao, W., Zhang, X., Pan, H. and Zhang, S., 2012. Biodegradation of nicosulfuron by the bacterium Serratia marcescens N80. *Journal of Environmental Science and Health, Part B*, *47*(3), pp.153-160. |
| 39 | Shewanella | *S. putrefaciens* | Fe (III) | DiChristina, T.J., Moore, C.M. and Haller, C.A., 2002. Dissimilatory Fe (III) and Mn (IV) reduction by Shewanella putrefaciens requires ferE, a homolog of the pulE (gspE) type II protein secretion gene. *Journal of bacteriology*, *184*(1), pp.142-151. |
| 40 | Sphingobium | *S. baderi* | Clodinafop propargyl | Li, Y., Chen, Q., Wang, C.H., Cai, S., He, J., Huang, X. and Li, S.P., 2013. Degradation of acetochlor by consortium of two bacterial strains and cloning of a novel amidase gene involved in acetochlor-degrading pathway. *Bioresource technology*, *148*, pp.628-631. |
|  |  | *S. quisquiliarum* | Clodinafop propargyl | Li, Y., Chen, Q., Wang, C.H., Cai, S., He, J., Huang, X. and Li, S.P., 2013. Degradation of acetochlor by consortium of two bacterial strains and cloning of a novel amidase gene involved in acetochlor-degrading pathway. *Bioresource technology*, *148*, pp.628-631. |
|  |  | *S. xenophagum* | Phenanthrene | Gran-Scheuch, A., Fuentes, E., Bravo, D.M., Jiménez, J.C. and Pérez-Donoso, J.M., 2017. Isolation and characterization of phenanthrene degrading bacteria from diesel fuel-contaminated Antarctic soils. *Frontiers in microbiology*, *8*, p.1634. |
| 41 | Sphingomonas | *S. haloaromaticamans* | Ortho-phenylphenol | Perruchon, C., Vasileiadis, S., Rousidou, C., Papadopoulou, E.S., Tanou, G., Samiotaki, M., Garagounis, C., Molassiotis, A., Papadopoulou, K.K. and Karpouzas, D.G., 2017. Metabolic pathway and cell adaptation mechanisms revealed through genomic, proteomic and transcription analysis of a Sphingomonas haloaromaticamans strain degrading ortho-phenylphenol. *Scientific reports*, *7*(1), p.6449. |
|  |  | *S. paucimobilis* | Methyl parathion | Lan, W.S., Lu, T.K., Qin, Z.F., Shi, X.J., Wang, J.J., Hu, Y.F., Chen, B., Zhu, Y.H. and Liu, Z., 2014. Genetically modified microorganism Spingomonas paucimobilis UT26 for simultaneously degradation of methyl-parathion and γ-hexachlorocyclohexane. *Ecotoxicology*, *23*(5), pp.840-850. |
| 42 | Stenotrophomonas | *S. acidaminiphila* | Bensulfuron-methyl | Lü, Z., Sang, L., Li, Z. and Min, H., 2009. Catalase and superoxide dismutase activities in a Stenotrophomonas maltophilia WZ2 resistant to herbicide pollution. *Ecotoxicology and environmental safety*, *72*(1), pp.136-143. |
|  |  | *S. maltophilia* | Chlorimuron | Zang, H., Yu, Q., Lv, T., Cheng, Y., Feng, L., Cheng, X. and Li, C., 2016. Insights into the degradation of chlorimuron-ethyl by Stenotrophomonas maltophilia D310-3. *Chemosphere*, *144*, pp.176-184. |
| 43 | Streptomyces | *S. albogriseolus* | Carbendazim | Arya, R. and K Sharma, A., 2016. Bioremediation of carbendazim, a benzimidazole fungicide using Brevibacillus borstelensis and Streptomyces albogriseolus together. *Current pharmaceutical biotechnology*, *17*(2), pp.185-189. |
|  |  | *S. aureus* | Deltamethrin | Chen, S., Lai, K., Li, Y., Hu, M., Zhang, Y. and Zeng, Y., 2011. Biodegradation of deltamethrin and its hydrolysis product 3-phenoxybenzaldehyde by a newly isolated Streptomyces aureus strain HP-S-01. *Applied microbiology and biotechnology*, *90*(4), pp.1471-1483. |
|  |  | *S. bikiniensis* | Alachlor | Sette, L.D., De Oliveira, V.M. and Manfio, G.P., 2005. Isolation and characterization of alachlor-degrading actinomycetes from soil. *Antonie van Leeuwenhoek*, *87*(2), pp.81-89. |
|  |  | *S. galbus* | Alachlor | Sette, L.D., De Oliveira, V.M. and Manfio, G.P., 2005. Isolation and characterization of alachlor-degrading actinomycetes from soil. *Antonie van Leeuwenhoek*, *87*(2), pp.81-89. |
| 44 | Variovorax | *V. boronicumulans* | Thiacloprid | Zhang, H.J., Zhou, Q.W., Zhou, G.C., Cao, Y.M., Dai, Y.J., Ji, W.W., Shang, G.D. and Yuan, S., 2011. Biotransformation of the neonicotinoid insecticide thiacloprid by the bacterium Variovorax boronicumulans strain J1 and mediation of the major metabolic pathway by nitrile hydratase. *Journal of agricultural and food chemistry*, *60*(1), pp.153-159.  Liu, Z.H., Cao, Y.M., Zhou, Q.W., Guo, K., Ge, F., Hou, J.Y., Hu, S.Y., Yuan, S. and Dai, Y.J., 2013. Acrylamide biodegradation ability and plant growth-promoting properties of Variovoraxboronicumulans CGMCC 4969. *Biodegradation*, *24*(6), pp.855-864. |
| 45 | Yersinia | *Y. frederiksenii* | Permethrin | Lee, S., Gan, J., Kim, J.S., Kabashima, J.N. and Crowley, D.E., 2004. Microbial transformation of pyrethroid insecticides in aqueous and sediment phases. *Environmental Toxicology and Chemistry: An International Journal*, *23*(1), pp.1-6. |

**Supplementary Table S5**. Evidence of pollutant degrading fungal species mentioned in present study

| **Sl. No** | **Genus** | **Species** | **Target pollutant** | **References** |
| --- | --- | --- | --- | --- |
| 1 | Aspergillus | *A. flavus* | Pb, Cd, Cr and Ni | Thippeswamy, B., Shivakumar, C.K. and Krishnappa, M., 2012. Bioaccumulation potential of Aspergillus niger and Aspergillus flavus for removal of heavy metals from paper mill effluent. *Journal of Environmental Biology*, *33*(6), p.1063.  Joshi, P.K., Swarup, A., Maheshwari, S., Kumar, R. and Singh, N., 2011. Bioremediation of heavy metals in liquid media through fungi isolated from contaminated sources. *Indian journal of microbiology*, *51*(4), pp.482-487. |
|  |  | *A. nidulans* | As | Maheswari, S. and Murugesan, A.G., 2009. Remediation of arsenic in soil by Aspergillus nidulans isolated from an arsenic‐contaminated site. *Environmental technology*, *30*(9), pp.921-926. |
|  |  | *A. niger* | Metalaxyl, Petroleum | Massoud, A.H., Derbalah, A.S. and Belal, E.S.B., 2008. Microbial detoxification of metalaxyl in aquatic system. *Journal of Environmental Sciences*, *20*(3), pp.262-267.  Hasan, I., 2014. Biodegradation of kerosene by Aspergillus niger and Rhizopus stolonifer. *Appl. Environ. Microbiol*, *2*(1), pp.31-36. |
|  |  | *A. oryzae* | Dye (Procion Red HE7B and Procion Violet H3R) | Corso, C.R. and De Almeida, A.C.M., 2009. Bioremediation of dyes in textile effluents by Aspergillus oryzae. *Microbial ecology*, *57*(2), p.384. |
|  |  | *A. terreus* | Endosulfan | Mukherjee, I. and Mittal, A., 2005. Bioremediation of endosulfan using Aspergillus terreus and Cladosporium oxysporum. *Bulletin of environmental contamination and toxicology*, *75*(5), pp.1034-1040. |
|  |  | *A. versicolor* | Hg | Du, F., Liu, Q., Wang, H. and Ng, T., 2014. Purification an α-galactosidase from Coriolus versicolor with acid-resistant and good degradation ability on raffinose family oligosaccharides. *World Journal of Microbiology and Biotechnology*, *30*(4), pp.1261-1267. |
| 2 | Bjerkandera | *B. adusta* | HCH | Quintero, J.C., Lu-Chau, T.A., Moreira, M.T., Feijoo, G. and Lema, J.M., 2007. Bioremediation of HCH present in soil by the white-rot fungus Bjerkandera adusta in a slurry batch bioreactor. *International Biodeterioration & Biodegradation*, *60*(4), pp.319-326. |
| 3 | Clonostachys | *C. rosea* | Heavy metal | Cecchi, G., Marescotti, P., Di Piazza, S. and Zotti, M., 2017. Native fungi as metal remediators: silver myco-accumulation from metal contaminated waste-rock dumps (Libiola Mine, Italy). *Journal of Environmental Science and Health, Part B*, *52*(3), pp.191-195. |
| 4 | Coprinus | *C. comatus* | Cd, Endosulphan | Wang, Y., Zhang, B., Chen, N., Wang, C., Feng, S. and Xu, H., 2018. Combined bioremediation of soil co-contaminated with cadmium and endosulfan by Pleurotus eryngii and Coprinus comatus. *Journal of soils and sediments*, *18*(6), pp.2136-2147. |
| 5 | Exophiala | *E. xenobiotica* | Toluene | Isola, D., Selbmann, L., de Hoog, G.S., Fenice, M., Onofri, S., Prenafeta-Boldú, F.X. and Zucconi, L., 2013. Isolation and screening of black fungi as degraders of volatile aromatic hydrocarbons. *Mycopathologia*, *175*(5-6), pp.369-379. |
| 6 | Fusarium | *F. solani* | DDT | Mitra, J., Mukherjee, P.K., Kale, S.P. and Murthy, N.B.K., 2001. Bioremediation of DDT in soil by genetically improved strains of soil fungus Fusarium solani. *Biodegradation*, *12*(4), pp.235-245. |
| 7 | Mucor | *M. racemosus* | Polycyclic aromatic hydrocarbon | Balaji, V., Arulazhagan, P. and Ebenezer, P., 2014. Enzymatic bioremediation of polyaromatic hydrocarbons by fungal consortia enriched from petroleum contaminated soil and oil seeds. *Journal of environmental biology*, *35*(3), pp.521-529. |
| 8 | Penicillium | *P. crustosum* | Pyrene | Ravelet, C., Krivobok, S., Sage, L. and Steiman, R., 2000. Biodegradation of pyrene by sediment fungi. *Chemosphere*, *40*(5), pp.557-563. |
| 9 | Phanerochaete | *P.chrysosporium* | Chlorinated organic compounds | Bumpus, J.A. and Aust, S.D., 1987. Biodegradation of chlorinated organic compounds by Phanerochaete chrysosporium, a wood-rotting fungus. *Solvent Hazardous waste problems: learning from dioxins. Washington DC: American Chemistry Society*, pp.340-349. |
|  |  | *P. sordida* | Clothianidin | Mori, T., Wang, J., Tanaka, Y., Nagai, K., Kawagishi, H. and Hirai, H., 2017. Bioremediation of the neonicotinoid insecticide clothianidin by the white-rot fungus Phanerochaete sordida. *Journal of hazardous materials*, *321*, pp.586-590. |
| 10 | Pleurotus | *P. eryngii* | Cd and endosulfan | Wang, Y., Zhang, B., Chen, N., Wang, C., Feng, S. and Xu, H., 2018. Combined bioremediation of soil co-contaminated with cadmium and endosulfan by Pleurotus eryngii and Coprinus comatus. *Journal of soils and sediments*, *18*(6), pp.2136-2147. |
|  |  | *P. ostreatus* | Creosote | García-Delgado, C., Alfaro-Barta, I. and Eymar, E., 2015. Combination of biochar amendment and mycoremediation for polycyclic aromatic hydrocarbons immobilization and biodegradation in creosote-contaminated soil. *Journal of hazardous materials*, *285*, pp.259-266.  Byss, M., Elhottová, D., Tříska, J. and Baldrian, P., 2008. Fungal bioremediation of the creosote-contaminated soil: influence of Pleurotus ostreatus and Irpex lacteus on polycyclic aromatic hydrocarbons removal and soil microbial community composition in the laboratory-scale study. *Chemosphere*, *73*(9), pp.1518-1523. |
| 11 | Rhizopus | *R. oryzae* | Heavy metal and dye | Mishra, A. and Malik, A., 2014. Novel fungal consortium for bioremediation of metals and dyes from mixed waste stream. *Bioresource technology*, *171*, pp.217-226. |
|  |  | *R. stolonifer* | Petroleum | Hasan, I., 2014. Biodegradation of kerosene by Aspergillus niger and Rhizopus stolonifer. *Appl. Environ. Microbiol*, *2*(1), pp.31-36. |
| 12 | Trametes | *T. hirsuta* IBB 450 | Polycyclic aromatic hydrocarbon | Batista-García, R.A., Kumar, V.V., Ariste, A., Tovar-Herrera, O.E., Savary, O., Peidro-Guzmán, H., González-Abradelo, D., Jackson, S.A., Dobson, A.D., del Rayo Sánchez-Carbente, M. and Folch-Mallol, J.L., 2017. Simple screening protocol for identification of potential mycoremediation tools for the elimination of polycyclic aromatic hydrocarbons and phenols from hyperalkalophile industrial effluents. *Journal of environmental management*, *198*, pp.1-11. |
|  |  | *T. versicolor* | Phenolic compounds | Udayasoorian, C. and Prabu, P.C., 2005. Biodegradation of phenols by ligninolytic fungus Trametes versicolor. *J. Biol. Sci*, *5*(5), pp.558-561. |
| 13 | Trichoderma | *T. harzianum* | Phenolic compounds | Singh, U.B., Singh, S., Malviya, D., Karthikeyan, N., Imran, M., Chaurasia, R., Alam, M., Singh, P., Sarma, B.K., Rai, J.P. and Damodaran, T., 2019. Integration of anti-penetrant tricyclazole, signaling molecule salicylic acid and root associated Pseudomonas fluorescens enhances suppression of Bipolaris sorokiniana in bread wheat (Triticum aestivum L.). *Journal of Plant Pathology*, pp.1-12. |
